# Comparison of radical-driven technologies applied for paraben mixture degradation: mechanism, biodegradability, toxicity and cost assessment

**DOI:** 10.1007/s11356-019-06703-9

**Published:** 2019-11-20

**Authors:** Marta Gmurek, João F. Gomes, Rui C. Martins, Rosa M. Quinta-Ferreira

**Affiliations:** 1 Chemical Process Engineering and Forest Products Research Centre (CIEPQPF), Department of Chemical Engineering, Faculty of Sciences and Technology, University of Coimbra, Coimbra, Portugal; 2 Faculty of Process and Environmental Engineering, Department of Bioprocess Engineering, Lodz University of Technology, Lodz, Poland

**Keywords:** Ozonation, Photocatalytic ozonation, Photocatalytic oxidation, Advanced oxidation processes, Paraben, Toxicity

## Abstract

Parabens (esters of *p*-hydroxybenzoic acid) are xenobiosis belonging to endocrine disruptors and commonly used as a preservative in cosmetics, food, pharmaceutical, and personal care products. Their wide use is leading to their appearance in water and wastewater in the range from ng/L to mg/L. In fact, the toxicity of benzylparaben is comparable to bisphenol A. Therefore, it is important to find not only effective but also ecofriendly methods for their removal from aqueous environment since the traditional wastewater treatment approaches are ineffective. Herein, for the first time, such extended comparison of several radical-driven technologies for paraben mixture degradation is presented. The detailed evaluation included (1) comparison of ozone and hydroxyl peroxide processes; (2) comparison of catalytic and photocatalytic processes (including photocatalytic ozonation); (3) characterisation of catalysts using SEM, XRD, DRS, XPS techniques and BET isotherm; (4) mineralisation, biodegradability and toxicity assessment; and (5) cost assessment. O_3_, H_2_O_2_/Fe^2+^, H_2_O_2_/UVC, O_3_/H_2_O_2_, O_3_/UVA, O_3_/H_2_O_2_/UVA, UVA/catalyst, O_3_/catalyst and O_3_/UVA/catalyst were selected from advanced oxidation processes to degrade parabens as well as to decrease its toxicity towards *Aliivibrio fischeri*, *Corbicula fluminea* and *Lepidium sativum*. Research was focused on the photocatalytic process involving visible light (UVA and natural sunlight) and TiO_2_ catalysts modified by different metals (Ag, Pt, Pd, Au). Photocatalytic oxidation showed the lowest efficiency, while in combining ozone with catalysis and photocatalysis process, degradation efficiency and toxicity removal were improved. Photocatalytic ozonation slightly improved degradation efficiency but appreciably decreased transferred ozone dose (TOD). Results indicate that the degradation pathway is different, or different transformation products (TPs) could be formed, despite that the hydroxyl radicals are the main oxidant.

**Electronic supplementary material:**

The online version of this article (10.1007/s11356-019-06703-9) contains supplementary material, which is available to authorized users.

**Figure:**
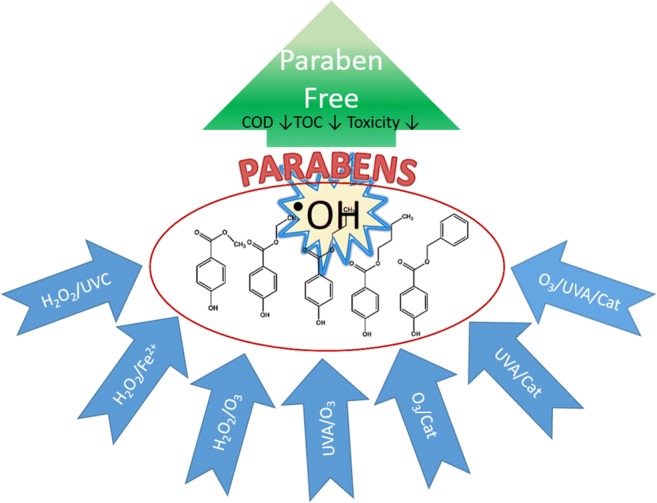
Graphical abstract

## Introduction

Xenobiotics are organic compounds foreign to living organisms, which do not occur in the form of neither primary nor secondary metabolites. While some xenobiotics are natural compounds, most of them are from anthropogenic source (Hashmi et al. 2017; Yang et al. 2017). They are included in personal protective equipment or pharmaceuticals, referred as pharmaceuticals and personal care products (PPCPs), whose presence in water reservoirs is a serious environmental problem (Yang et al. 2017). The compounds belonging to the PPCP group (e.g. parabens) have different physicochemical properties, which make them difficult to detect and remove from wastewater and natural environment (Nakada et al. 2007; Yang et al. 2017; Wang et al. 2018). Many of xenobiotics have been classified as compounds that disrupt the hormonal balance of the body (xenooestrogenic or endocrine compounds) called endocrine-disrupting compounds (EDCs) (Nakada et al. 2007; Esplugas et al. 2007; Giulivo et al. 2016; Wang and Wang 2016). Numerous studies have confirmed the harmful effect of xenobiotics on disturbing the hormonal balance of organisms, hepatotoxic effects damaging liver cells and the possibility of producing free radicals which cause oxidative stress (Vilela et al. 2018).

Xenobiotics are an interesting research object since they are a group of compounds with significant toxic effects. In addition, in trace amounts, they occur in the aquatic environment. However, even at very low concentrations (ppb or ppm), these compounds are dangerous for the environment, and contaminated water causes a threat to people and also affects the condition of animal populations and plants (Gu et al. 2002). Unfortunately, the still growing consumerism means that the presence of xenobiotics in the lives of people, animals and the environment is inevitable. It is known that the removal of EDCs in wastewater treatment plants using conventional methods is insufficient (Nakada et al. 2007; Esplugas et al. 2007; Wang and Wang 2016; Gmurek et al. 2017; Yang et al. 2017; Wang et al. 2018). Poor efficiency is probably related to physical and chemical properties as well as its toxicity. Considering the product cycle, these compounds in unmetabolised form were found in wastewater, from where they get mainly to water reservoirs (e.g. used pesticides as a result of runoff from agricultural areas get into surface waters). Consequently, they are detected in drinking water from which they can be absorbed into the body.

Parabens (esters of *p*-hydroxybenzoic acid) represent xenobiotics, PPCPs and EDC groups commonly used as preservative in cosmetics and personal care products, food, pharmaceuticals, etc. Although it was found that butylparaben can be synthesised by marine bacteria (Peng et al. 2006), only produced synthetically commercial parabens are used for industrial purpose (Juliano et al. 2017). Furthermore, for the last 10 years, the EU regulation about paraben content in cosmetic products has been restricted. According to Regulation (EU) No. 358/2014 (Juliano et al. 2017), isopropylparaben, isobutylparaben, phenylparaben, benzylparaben and pentylparaben are prohibited in cosmetic products. As reported by Regulation (EU) No. 1004/2014 (Juliano et al. 2017), propylparaben and butylparaben (also their isoforms and salts) were banned from cosmetic products for children under 3 years of age, based on those substances’ potential endocrine activity. Additionally, the maximum concentration allowed for these two parabens was reduced in the cosmetic products for adults and kids above 3 years of age (Juliano et al. 2017). Parabens can be considered as endocrine disruptors and carcinogens (Giulivo et al. 2016; Juliano et al. 2017). Moreover, the toxicity and oestrogenicity of benzylparaben is comparable to that of bisphenol A (Martins et al. 2016). Parabens were detected in influents from WWTPs (even at concentrations of mg/L), and after the treatment in effluents, their concentrations were still high (including BzP) (Canosa et al. 2006; Haman et al. 2015; Marta-Sanchez et al. 2018; Gomes et al. 2018a). The presence of paraben was recorded in natural water in Japan, the UK, Portugal, Switzerland, Belgium, China, Spain and the USA in the range of 0.1–676 ng/L (Jonkers et al. 2010; Haman et al. 2015). Some of them were detected in drinking water in the concentration of ng/L in Germany, Spain or the USA (Haman et al. 2015; Marta-Sanchez et al. 2018). Swimming pools are also contaminated by parabens as well as sediments (Haman et al. 2015; Lempart et al. 2018). Due to the characteristics of parabens, which have a negative impact on human and animal populations, a method of their degradation is sought, because nature also fails on removing them through biological process. Moreover, the complex structure causes their insufficient removal in the wastewater treatment process, and as a result of which, they accumulate in living organisms. Until now, commonly applied methods of wastewater treatment or water treatment are ineffective, and a significant amount of these compounds is presented in water. Therefore, it is important to develop efficient methods for eliminating them from wastewater.

Based on literature data, the most effective method of removing them is chemical oxidation using reactive oxygen species generated using ultraviolet (UV) or visible (Vis) radiation or ozone (Nakada et al. 2007; Gottschalk et al. 2010; Nawrocki and Kasprzyk-Hordern 2010; Tay et al. 2010; Gmurek et al. 2015, 2017; Wang and Wang 2016; Pipolo et al. 2017; Gomes et al. 2017a; Wang et al. 2018; Foszpańczyk et al. 2018). These methods often allow to obtain products more susceptible to biodegradation. Studies focusing on photodegradation processes of compounds contaminating the aquatic environment are mostly aimed at intensifying the degradation process and obtaining non-toxic phototransformation products. The preferred direction of the development of these methods is the possibility of using renewable energy in the form of solar radiation. Many of the known photochemical methods belong to the group of advanced oxidation processes (AOPs). Lately, AOPs are the most applicable treatment methods for removing hazardous water contaminants, despite that lots of them have a limitation due to optimal pH parameter. O_3_ has two pathways of degradation: in acidic pH, direct oxidation via O_3_, while in alkaline pH, indirect oxidation via ^•^OH (Glaze 1986; Glaze et al. 1987; Lucas et al. 2010). H_2_O_2_/Fe^2+^ has an optimum effectivity at pH ≈ 3, particularly due to the precipitation of ferric oxyhydroxide at higher pH value (Ikhlaq et al. 2018, 2019). However, alkaline environment is the favourable pH for most AOPs (Glaze et al. 1987; Lucas et al. 2010; Xiao et al. 2015; Boczkaj and Fernandes 2017; Shahzad Munir et al. 2019; Asgari et al. 2019; Ikhlaq et al. 2019). In fact, the AOPs are aimed to be applied as tertiary systems for the removal of hazardous water contaminants from municipal wastewater treatment plants (Gmurek et al. 2017; Wang et al. 2018; Miklos et al. 2018; Soriano-Molina et al. 2018). Usually, these effluents are treated in secondary reactors based in biologic systems that require neutral pH conditions for proper operation. Therefore, it is interesting that the following treatment technologies also operate under those conditions (further pH adjustment is not necessary) which reduce operating costs, even in case of Fenton process, however, with the disadvantage of iron precipitation (Soriano-Molina et al. 2018). The second important issue in case of AOP is the light source. Unfortunately, the visible light can be applied as an effective light source only for few AOPs. Mostly, they focus on the application of UV radiation with various combinations of other oxidants (H_2_O_2_, O_3_, Fenton reagent, TiO_2_). Even though processes using UV radiation are energy-consuming, these are still widely used due to their high efficiency and rate of degradation process. The use of UV radiation also allows for process control (constant radiation conditions). However, it should be borne in mind that the use of additional oxidants may cause further environmental problems (for example, the resulting sediment during the photo-Fenton process). Thus, processes such as photolysis using UV, UV/H_2_O_2_, TiO_2_/UV, O_3_/UV and TiO_2_/O_3_/UV can form the foundations of research works using photochemical processes, allowing determination of quantum yields or kinetic constants. Nowadays, the best catalyst is sought; for example, the addition of a second semiconductor will shift the spectrum absorbed by the semiconductor towards the visible light. Moreover, photocatalyst surface modifications by metals are affected by changing the distribution of electrons on the properties of the semiconductor, which increases the photodegradation activity (Nawrocki and Kasprzyk-Hordern 2010; Grabowska et al. 2016; Gmurek et al. 2017; Gomes et al. 2017b; Foszpańczyk et al. 2018).

Bearing in mind the problem associated with parabens, it is important to find not only effective but also ecofriendly methods for their removal from aqueous environment. From all well-known water and wastewater treatment technologies, AOPs are not only important, efficient and ecofriendly but also promising methods that allow to remove persistent aqueous contaminants.

To the best of our knowledge, this is the first time that such extended comparison of several AOPs for the mixture of water pollutant degradation has been presented. The detailed evaluation included (1) comparison of ozone and hydroxyl peroxide processes; (2) comparison of catalytic and photocatalytic processes (including photocatalytic ozonation); (3) characterisation of catalysts using SEM, X-ray diffraction (XRD), DRS, XPS techniques and BET isotherm; (4) mineralisation, biodegradability and toxicity assessment; and (5) cost assessment. H_2_O_2_/Fe^2+^, H_2_O_2_/UVC, O_3_, O_3_/H_2_O_2_, O_3_/UVA, O_3_/H_2_O_2_/UVA, UVA/catalyst (UVA/Cat), O_3_/Cat and O_3_/UVA/catalyst (O_3_/UVA/Cat) were selected from AOPs to degrade parabens from aqueous environment. As photocatalysts, noble metals (Pt, Pd, Au, Ag) that modified TiO_2_ were applied and compared with pure TiO_2_. For photocatalytic oxidation, the comparison of UVA light and natural sunlight was made. The toxicity assessment before and after treatment was evaluated by *Vibrio fischeri* luminescence inhibition and the freshwater Asian clam *Corbicula fluminea* mortality. Moreover, the phytotoxicity test using *Lepidium sativum* was performed.

## Materials and methods

### Chemicals and reagents

A solution of five parabens [methylparaben (MP), ethylparaben (EP), propylparaben (PP), butylparaben (BuP) and benzylparaben (BeP)] was used as a model effluent. The pollutant solutions were prepared with Milli-Q (MQ) water using 10 mg/L of each compound. A commercial form of TiO_2_ (P25, crystalline composition of 80% anatase and 20% rutile, with a surface area of 50 m^2^/g) was obtained from Evonik (Germany). 2-Propanol (for analysis) was purchased at Merck (Germany). All solvents [HPLC water (VWR), acetonitrile and formic acid (both from Merck)] were analytical grade (≥ 99%). Experimental solutions were prepared using ultrapure water.

### Preparation of photocatalysts

Two methods of TiO_2_ modification were used. TiO_2_-Pt, TiO_2_-Pd and TiO_2_-Ag were prepared by UV reduction of Pt^4+^, Pd^2+^ and Ag^2+^ used as chloride salts, in the TiO_2_ (P25) suspension, while TiO_2_-Au was prepared by the sol–gel method, according to Gomes et al. (2017a). In photodeposition method, an aqueous solution of isopropanol containing H_2_PtCl_6_ (0.5 wt%) or PdCl_2_ (0.5 wt%) was degassed with nitrogen and irradiated by UV–Vis light (1000 W Xe lamp) for 6 h. For TiO_2_-Ag preparation, an aqueous solution of ethanol containing AgNO_3_ (0.5 wt%) was degassed with nitrogen and irradiated by UV–Vis light (1000 W Xe lamp) for 100 min. All catalysts prepared by the photodeposition method were separated by centrifugation and dried at 65–120 °C for 12 h. In the sol–gel method, titanium(IV) isopropoxide (TIP), known as the titanium source for the anatase-type TiO_2_, was used for TiO_2_-Au preparation. TIP was mixed with AuCl_4_K (0.5 wt%), methanol and distilled water. The solution was stirred at room temperature for 2 h, followed by a 24-h thermal treatment (45 °C) and calcinations at 400 °C for 2 h.

### Characterisation of photocatalysts

The microstructural analysis of the surfaces was performed by TESCAN VEGA3 SBH - Easy Probe Scanning Electron Microscopy (SEM) with a tungsten-heated cathode. The SEM images were acquired with a working tension of 5 kV, using the secondary electron detector. The optical properties of the photocatalysts were characterised by an ultraviolet/visible diffuse reflectance spectrophotometer (Evolution 220; Thermo Scientific), in which BaSO_4_ was employed as the internal reflectance standard. The band gaps for all photocatalysts were determined using the Tauc plot method. The surface areas of catalysts Brunauer–Emmett–Teller were determined using nitrogen (− 196 °C) with an accelerated surface area and porosimetry analyser (ASAP 2000, Micrometrics). The crystal structure of photocatalysts was determined from XRD pattern measured in the range of 2*θ* = 20–80° using an X-ray diffractometer (X’Pert PRO-MPD, Philips) with Cu target (*λ* = 1.542 Å). The lattice parameters were estimated by the Le Bail method using FullProf package.

### Light sources

Irradiation experiments were performed using a UVC (254-nm) lamps, UVA (365-nm) lamps and natural sunlight (in Poland as well as in Portugal). Six low-pressure mercury lamps (USHIO, model G8T5 Hg; 7.2 W each) with a maximum emission of 254 nm (88.6%) were used as a UVC source (*E*_0_ = 1.06 × 10^−5^ E/Ls; 31.8 W/m^2^). Three lamps made of blacklight blue glass, which transmits UVA radiation (Philips TL 6 W BLB; tube diameter of 16 mm), with the maximum emission of 365 nm were used as UVA source (*E*_0_ = 5.75 × 10^−7^ E/Ls; 8.9 W/m^2^). The natural sunlight was applied, and the experiments were conducted in the sunny days (in July 2016 and May 2018) with the average irradiance of 340 W//m^2^, corresponding to the photon flux rate *E*_0_ = 2.60 × 10^−4^ E/Ls.

The experiments with UVC lamps were carried out in quartz test tubes (10 mL and an average optical path length of 0.85 cm) placed between two exposure panels using a merry-go-round device.

The experiments with a UVA lamps were carried out in a 2-L reactor with internal lamp source. The experiments with sunlight were carried out in a cylindrical borosilicate reactor (0.5 L) equipped with a compound parabolic concentrator (CPC) collector. All reactors set up are presented in Fig. S1.

During the experiments, the light exposition was measured with an Oceans Optics USB 4000 fibre optic spectrometer with an approximate resolution of 0.4 nm. All the experiments were performed twice. Results are presented as a mean value of single experiments.

### Experimental procedure

#### H_2_O_2_/Fe^2+^ process

Reaction was carried out in a batch-stirred glass reactor. Ferrous ion (Fe^2+^) was added in the form of iron sulphate, and the reaction started when the desired amount of H_2_O_2_ was injected. pH was left to run freely (at the beginning, 6 ± 0.7, and after 120 min of reaction, the final pH value falls to 3.2 ± 0.3). A detailed procedure and an experimental set-up were previously published in the study of Martins et al. (2016).

#### H_2_O_2_/UVC

The reaction set-up was composed by quartz test tubes (10 mL) placed between two exposure panels using a merry-go-round device at a distance of 15 cm from its centre. Each panel was composed by three low-pressure mercury lamps (7.2 W each) with a maximum emission for 254 nm. The pH of the reaction medium was 7, adjusted by a phosphate buffer. A detailed procedure and an experimental set-up were previously published in the study of Gmurek et al. (2015).

#### O_3_-based technology

Two kinds of equipment were used for ozone-based technology. The first one was a semi-batch reactor of 500 mL which was used to perform ozonation experiments (single ozonation (O_3_) and O_3_/H_2_O_2_). Ozone was produced in situ by an ozone generator that was fed by a pure oxygen stream (99.9%) supplied by Praxair. Ozone concentration in the gas entering ([O_3_]^in^) and leaving ([O_3_]^out^) the reactor was measured by a gas ozone meter. To ensure chemical regime, the reactor was agitated at the approximate speed of 700 rpm by means of a magnetic stirrer. The pH of the reaction medium was 7, adjusted by a phosphate buffer. A detailed procedure and an experimental set-up were previously published in the study of Pipolo et al. (2017). The second one was a 2-L glass reactor with the possibility of using 3 UVA lamps which was used for O_3_, O_3_/UVA, UVA/Cat and O_3_/Cat O_3_/UVA/Cat experiments. The stirring speed was previously optimised at 700 rpm to ensure chemical regime. Along the reaction, samples were withdrawn and immediately centrifuged at 3500 rpm to remove the photocatalyst. pH was left to run freely (at the beginning, 5.7). Ozone was produced from pure oxygen stream (99.9%), and the inlet ([O_3_]^in^) and outlet ([O_3_]^out^) ozone concentrations were measured by ozone analysers. A detailed procedure and an experimental set-up were previously published (Gomes et al. 2017a, b, c, d).

The photocatalytic experiments were carried out with 0.07 g/L of the photocatalyst. To test the adsorption capacity of photocatalyst, it was placed in the reactor with the solution of parabens 5 min before ozone being fed and turning on the light irradiation. During the degradation experiments, the reactor was bubbled by pure oxygen stream (99.9%, Praxair, 0.2 L/min).

All process parameters are collected in Table S1.

### Analytical methods

The concentration of paraben in the mixture was followed by HPLC (UFLC, Shimadzu) with a C18 (SiliaChrom) column at 40 °C. The detection wavelength was 255 nm. The injection volume was 20 μL, and the mobile phase consisting a mixture of 50:50 methanol:acidic water (0.1% orthophosphoric acid) was used with the flow rate of 0.5 mL/min.

Chemical oxygen demand (COD) was measured according to the standard method 5220D using a calibration curve obtained for potassium hydrogen phthalate (Panreac). The absorbance of the samples was measured at 445 nm (WTW photoLab S6 photometer) after 2 h of digestion at 150 °C (ECO25; VELP Scientifica).

The absorption spectra of parabens were measured in a 1-cm quartz cell on a Unicam spectrophotometer.

### Toxicity assessment

The toxicity assessment was investigated by luminescence inhibition tests with *A. fischeri* using a LUMIStox 300 apparatus. The bioassays with *C. fluminea* were conducted using adult clams. Before clams were used as a bioindicator, they were maintained at least 1 week under continuous aeration at constant temperature and photoperiod (16-h light and 8-h dark) prior to use. The laboratory culture water was fully renewed once a week. Mortality tests were conducted under static conditions (Gomes et al. 2014). Blank controls with dechlorinated municipal water were applied along with a dilution series of untreated and treated model effluents with exposure lasting 72 h. *L. sativum* was considered as a bioindicator in the phytotoxicity test. Toxic effects, expressed as germination index (GI), were calculated according to Gomes et al. (2017a). Moreover, the level of the toxic impact on *L. sativum* was assessed by the method suggested by Wang (1992).

## Results

### Preliminary study

**Fig. 1:**
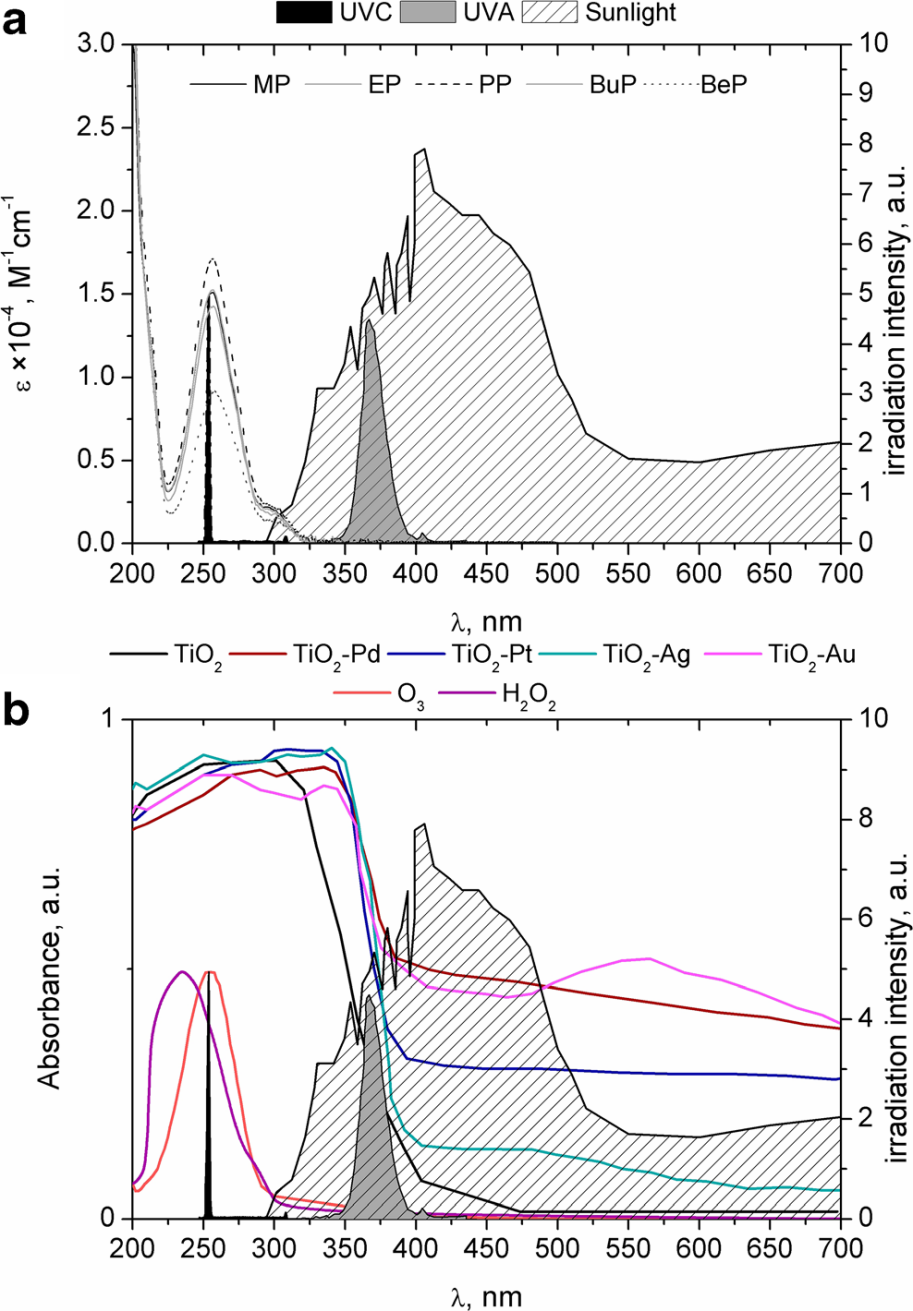
Absorption spectra of parabens (**a**) and photocatalysts, ozone and hydrogen peroxide (**b**) on the background of emission spectra of UVC and UVA lamps as well as sunlight

UV radiation is very often used in photodegradation processes. However, the use of UV radiation alone in purification or treatment processes is uneconomical (Gmurek et al. 2017). Most often, monochrome radiation with a wavelength of 254 nm is used, which, in the case of most organic substances, overlaps with the maximum of their absorbance (Fig. 1). Unfortunately, UVC radiation in this range does not occur in the spectrum of solar radiation at the Earth’s surface. Thus, studies based on UVC (254 nm) techniques cannot be directly translated into processes occurring in the natural water environment. Nevertheless, these technologies are still very popular, especially in disinfection systems. Photochemical degradation techniques are also conducted using polychromatic UVA radiation, which falls within the solar spectrum (Fig. 1). Most often, UV radiation is combined with other oxidants, which leads to the generation of hydroxyl radicals (•OH) (Gmurek et al. 2017). It is known that the use of •OH generated as an oxidant in AOPs leads to very rapid degradation of compounds and high mineralisation. Such high degradation efficiency is obtained as a result of the low selectivity of these oxidants. However, to generate hydroxyl radicals, a lot of energy and an additional oxidiser are needed. On the other hand, the use of less energetic visible radiation is associated with a decidedly lower reaction rate, i.e. a longer photodegradation time. As it was mentioned above, the modification of photocatalysts can improve their photoreactivity in the range of visible light. As can be seen in Fig. 1, all investigated photocatalysts exhibit very strong absorption below 400 nm that can be explained by the property of titanium to absorb UV light. Above 400 nm in the visible light region, the best absorption properties have been shown by TiO_2_-Au and TiO_2_-Pd, while the worst absorption properties have been shown by TiO_2_-Ag. However, all modified photocatalyst are able to absorb visible light, which can be attributed to the surface plasmon resonance of noble metal nanoparticles (Leong et al. 2015; Grabowska et al. 2016).

To evaluate the applicability of photodegradation processes to decontamination of parabens, the direct photolysis of their mixture under UVC and UVA lamps as well as sunlight has been compared. As it was expected, according to the results depicted in Fig. 1, almost complete removal after 1 h has been achieved when a UVC lamp was used (Fig. S2). Besides, negligible degradation was observed under UVA light, while natural sunlight has not let any degradation at all (Fig. S1).

### Comparison of ozone and H_2_O_2_ technologies

**Fig. 2:**
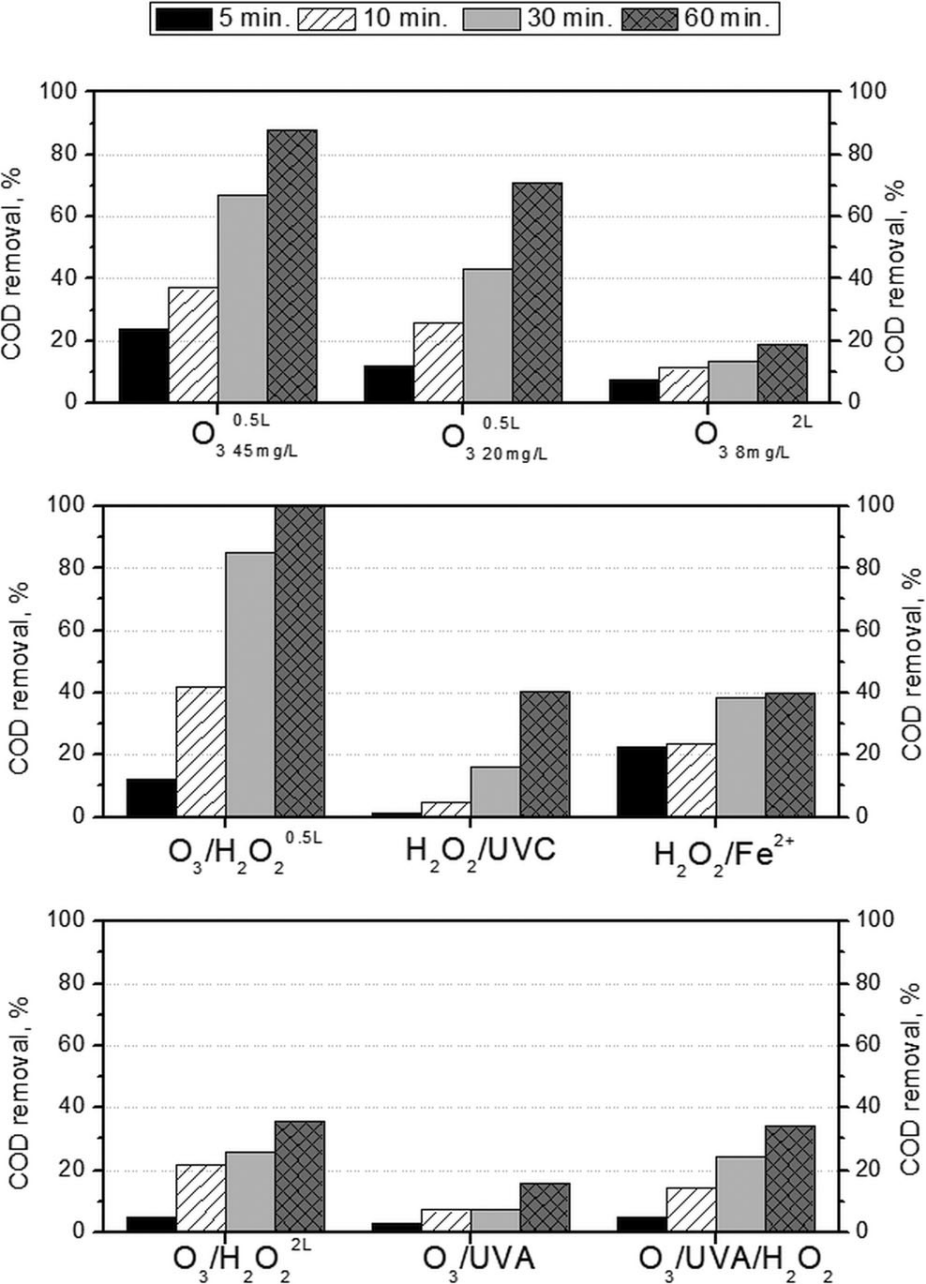
Comparison of COD removal by different AOPs

It is well known that the capable action of ozone, H_2_O_2_ and light improves the effectivity of decontamination. However, it should be noticed that the process parameters such as hydrodynamic conditions are significant. The synergetic effects are not always observed; moreover, a scavenging effect can be also expected, while some oxidants are added in extent. Furthermore, the decrease of initial concentrations is useful for the understanding of reaction mechanisms, but it is not sufficient for real effluents containing a mixture of organic compounds and no information is given about degradation of by-products. More detailed information is given by COD removal since the global organic matter removal is followed. As can be seen in Fig. 2, the process parameters are extremely important. Although all processes led to complete removal of initial concentration of parabens after 2 h (mostly resulted in 80% concentration reduction of parabens after 1 h), the COD removal is totally different for all of them.

The efficiency of ozone-based technology is highly dependent on pH, which has the ability to change the ozone oxidation pathway and dissolve ozone concentration (Hoigné and Bader 1976; Olak-Kucharczyk and Ledakowicz 2016; Pipolo et al. 2017; Asgari et al. 2019). In an acidic condition, molecular ozone pathway occurs; in neutral and alkaline environments, the hydroxyl radical pathway is promoted. Furthermore, O_3_ solubility increased with a decrease in pH (Biń 2006), affecting ozone concentration in liquid. It is well known that the ozone saturation is dependent not only on temperature but also on pH which can be effected by liquid constituents or impurities such as salinity and ionic strength (Biń 2006). If the AOP treatment is taken into consideration, two possible situations can occur: it can be supplemented for secondary effluents after biological treatment (pH close to neutral) or before biological treatment (effluents do not have to be buffered). Therefore, three reaction conditions during single ozonation have been investigated. At buffered neutral condition (pH ~ 7) in small volume reactor (500 mL), two ozone inlet gas concentrations were used (45 mg O_3_/L and 20 mg O_3_/L). While in non-buffered solution (2-L reactor), 8 mg O_3_/L was applied. In both cases, the mass transference resistances can be neglected (Gomes et al. 2017c; Pipolo et al. 2017). It is well known that hydroxyl radicals are more reactive than molecular ozone. Second-order rate constants for reaction with ^•^OH are 10^9^–10^10^ M^−1^/s order of magnitude (Tay et al. 2010; Gmurek et al. 2015), while the second-order rate constants for reaction of organic molecules with ozone are lower ~ 10^2^–10^3^ M^−1^/s or ~ 10^5^–10^6^ M^−1^/s in acidic or neutral condition, respectively (Tay et al. 2010; Olak-Kucharczyk and Ledakowicz 2016). Ozone dose is an important key parameter in determining the residual ozone concentration. However, the ozone decomposition increases with increasing pH. Therefore, at low pH, less ozone concentration is required because ozone lifetime is much longer than in neutral or alkaline environment (Biń 2006; Hansen et al. 2016).

As can be seen in Fig. 2, by single ozonation in buffered solution when the reaction occurs via ^•^OH, the COD removal was much higher than in not buffer solution. At the buffered solution, it was observed that with increasing ozone dosage, the reaction was accelerated. However, in the non-buffered solution, the ozone concentration was much lower, and the increase of it would be meaningless. The ozone pathways will always be slower than ^•^OH. In case when the pH was not adjusted (initial pH = 5.7 then decreased during the reaction), even when H_2_O_2_ or UVA was used, the effectivity has not increased significantly (Fig. 2). From integration of ozonation with the biological system point of view, if the initial concentration of contaminants was degraded, higher COD removal is not required, because biological treatment will be able to handle it.

However, application of single ozonation led to the problem of residual O_3_ in treated wastewater that does not allow for the immediate use of biological treatment after it (the remaining dissolved ozone must be let to be decomposed first before the biological reactor). This disadvantage can be overcome by the merging action of oxidants. It was mentioned that combining O_3_ with UV or H_2_O_2_ to enhance •OH generation is often more effective than O_3_ alone. The best results have been obtained for O_3_/H_2_O_2_ (0.5 L) treatment when the ^•^OH radical pathways occurred (pH = 7). Even the application of H_2_O_2_/UVC and Fenton reagent did not give better results (Fig. 2). It should be noticed that in the case of the Fenton process after the addition of iron sulphate and hydrogen peroxide, pH decreased from neutral to pH 3.6 [optimal condition according to Neyens and Baeyens 2003, Martins et al. 2016 and Ikhlaq et al. 2018]. The synergetic effect of H_2_O_2_ and O_3_ has been also observed in the case of non-buffered solution (Fig. 2).

The generation of •OH in O_3_/UV is initiated by the photolysis of O_3_ by UV (Gottschalk et al. 2010). The application of UVC (254 nm) enhances the oxidation potential due to the high molar extinction coefficient of O_3_ (*ε*_254_ = 3300 M^−1^/cm) (Gottschalk et al. 2010) (Fig. 1b). However, the major drawback of the ozone-based technology especially UV/O_3_ is generating high operational costs (the energy requirement of O_3_ production and UVC lamp equipment). The cheaper UVA lamp (365 nm) is not effective as a UVC source, because the ozone molecule is not absorbed in this region, so the •OH is not effectively produced. Therefore, the COD removal is even lower than in the case of single ozonation (Fig. 2). A better COD removal is achieved when O_3_/H_2_O_2_ was applied, especially when effectivity has been considered from the transferred ozone dose (TOD) point of view (Fig. S3). The action of O_3_ with H_2_O_2_ and UVA led to worse results than O_3_/H_2_O_2_ and lower COD removal and required more TOD (Fig. 2, Fig. S3). However, the O_3_/H_2_O_2_/UVA system exhibited higher COD removal in comparison to single ozonation and O_3_/UVA and required less TOD (Fig. 2, Fig. S3). Based on the results, it was shown that the effectivity of the process with respect to time as well as TOD must be considered.

### Photocatalytic processes

As shown in Fig. 1b, the photocatalysts with noble metals like Pd, Ag, Pt and Au effectively absorbed UVA as well as visible light. Moreover, it was proved that both of this light do not cause the photodegradation of parabens directly via photolysis (Fig. S1). TiO_2_-Pd and TiO_2_-Au exhibit the best absorption characteristics in the visible light range than TiO_2_-Pt and TiO_2_-Ag, while TiO_2_ has the worst ability to absorb visible light (Fig. 1b). That can be attributed to the width of the band gaps as shown in Table 1 and Fig. S4. Indirect/direct band gaps of pure TiO_2_ (3.27 eV/3.38 eV) were reduced to 3.00 eV/3.22 eV, 2.74 eV/3.23 eV, 2.59 eV/3.19 eV and 2.5 eV/3.13 eV for TiO_2_-Ag, TiO_2_-Pt, TiO_2_-Au and TiO_2_-Pd (all 0.5 wt%), respectively (Table 1). Since TiO_2_-Pd and TiO_2_-Au have the narrowest and similar band gaps (both direct and indirect), both perform in the Vis light range better, increasing the efficiency of the photocatalysis. The improvement in visible range absorption ability may be attributed to the surface plasmon resonance of noble metal nanoparticles. In Fig. 1b, in visible range, the maximum absorption peak due to localised surface plasmon resonance (LSPR) for TiO_2_-Au and TiO_2_-Ag can be observed (positioned in the range of 525–600 nm and 450–500 nm, respectively). It is well known that the photoactivity has also influenced the particle size, shape and particle distribution. Moreover, the sol–gel method gives ability to synthesise nanosized titania with high purity at low temperature (Xiao et al. 2015), while the photodeposition method allows a better particle size distribution control (Wenderich and Mul 2016). SEM images present the successfully deposited noble metals (Fig. S5). The modification by noble metal resulted in the homogeneous distribution of the Pd, Ag as well as Au particles on the catalyst surface. However, in the case of Pt and Au, particles were incorporated on the TiO_2_ surface in an irregular manner presenting higher agglomeration of particles or the size of particles. XRD measurement confirmed crystallographic forms after modification and showed that the anatase phase is predominant (Table S2). The results proved that surface modification with noble metals did not change significantly the basic structure of TiO_2_ (P25). Moreover, results indicated that titania, for the TiO_2_-Au nanoparticles, prepared by TIP, was present in the pure anatase structure. The size of anatase crystallites was the biggest for TiO_2_-Pd while the smallest for TiO_2_-Au. The BET analysis shows that the specific surface area was not significantly changed after modification (Table S2).

**Table 1:** Direct and indirect band gaps obtained using the Tauc plot (Fig. S3)

Band gap	TiO_2_ (eV)	TiO_2_-Ag (eV)	TiO_2_-Pt (eV)	TiO_2_-Au (eV)	TiO_2_-Pd (eV)
Direct	3.38	3.22	3.23	3.19	3.13
Indirect	3.27	3.00	2.74	2.59	2.5

**Fig. 3:**
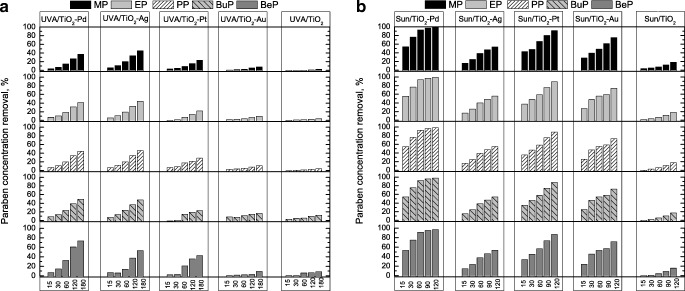
Photocatalytic oxidation of paraben mixture under UVA (**a**) and natural sunlight (**b**) during 15 min, 30 min, 60 min, 120 min and 180 min

As can be seen in Fig. 3a, the photocatalytic oxidation under UVA with TiO_2_ led to negligible paraben decontamination after 3 h. Unfortunately, the photocatalytic oxidation with TiO_2_ modified by noble metals has not caused the complete degradation of paraben concentration; therefore, the COD reduction exceeded 20%. In comparison to TiO_2_, only a slight improvement has been obtained when TiO_2_-Au was applied. The best results, but still not enough satisfactory, were obtained for TiO_2_-Pd and TiO_2_-Ag (Fig. 3a). The highest degradation was achieved for BeP (73% and 53% of BeP initial concentration for TiO_2_-Pd and TiO_2_-Ag, respectively). However, for the rest of parabens, the removal was below 50% of their concentration, while when COD removal is taken into account, only 19% and 17% were obtained after 3 h of the treatment for TiO_2_-Pd and TiO_2_-Ag, respectively. It was surprising that despite the low initial paraben degradation during TiO_2_-Pt photocatalytic treatment, 18% of COD removal was achieved. In case of TiO_2_ as well as TiO_2_-Au, the COD removal was around 10%.

**Fig. 4:**
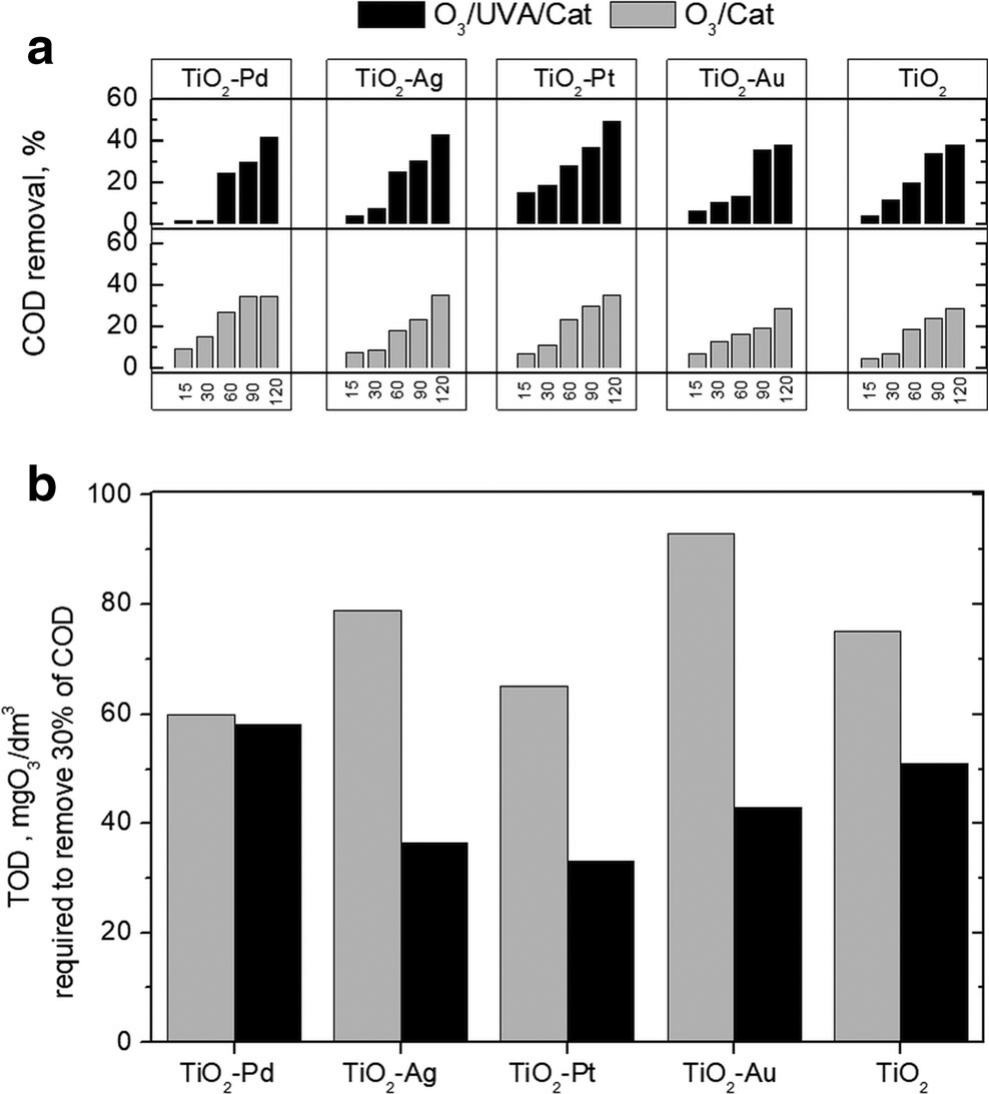
**a** Catalytic and photocatalytic ozonation of paraben mixture during 15 min, 30 min, 60 min, 90 min and 120 min. **b** TOD requirements for removing 30% of COD during catalytic and photocatalytic ozonation

Much better results have been obtained when natural sunlight was applied. Indeed, significant improvement was observed for all catalysts. Moreover, the use of TiO_2_-Pd led to total paraben degradation (Fig. 3b). Also, higher degradation was achieved in the case of TiO_2_-Pt rather than for TiO_2_-Ag, indicating that this photocatalyst has better visible light photocatalytic ability (Foszpańczyk et al. 2018). However, the highest improvement has been observed for TiO_2_-Au, proving that the modification by Au increases the visible light ability, which is in agreement with absorption spectra. Despite higher degradation under sunlight, the effectivity of photocatalytic processes was much below expectation. Therefore, the catalytic ozonation as well as the photocatalytic ozonation have been investigated (Fig. 4). As it was expected, the catalytic ozonation as well as the photocatalytic ozonation increased effectivity. The parabens were totally removed during the first 2 h of both treatments, leading to higher mineralisation than for O_3_ or O_3_/UVA. It is well known that the catalytic ozonation allows for the effective formation of hydroxyl radicals also at a low pH in contrast to single ozonation (Nawrocki and Kasprzyk-Hordern 2010). Furthermore, as expected, the photocatalytic ozonation led to higher COD removal. In case of 2 h of O_3_/Cat treatment, TiO_2_ and TiO_2_-Au exhibited similar effectivity (28% of COD removal). While the application of TiO_2_-Pt, TiO_2_-Ag and TiO_2_-Pd photocatalysts caused around 35% of COD removal. After 2 h of O_3_/UVA/Cat treatment, 38% of COD removal was achieved when TiO_2_ and Au-TiO_2_ were used, while 41%, 43% and 49% of COD were removed when TiO_2_-Pd, TiO_2_-Ag and TiO_2_-Pt, respectively, were applied. It was surprising that O_3_/H_2_O_2_ as well as O_3_/H_2_O_2_/UVA gave better COD removal (both above 33%) efficiency than O_3_/Cat and O_3_/UVA/Cat [after 1 h, 27% (TiO_2_-Pd) and 28% (TiO_2_-Pt), respectively] (Figs. 2 and 4). However, when TOD is taken into account, the requirements are much lower when catalytic ozonation and photocatalytic ozonation were considered (Fig. S3, Fig. 4b). As can be seen in Fig. 4b, the orders of the highest ozone-demanding catalysts are TiO_2_-Au < TiO_2_-Ag < TiO_2_ < TiO_2_-Pt < TiO_2_-Pd and TiO_2_-Pd < TiO_2_ < TiO_2_-Au < TiO_2_-Ag < TiO_2_-Pt during O_3_/Cat and O_3_/UVA/Cat, respectively. The differences can be related with ozone adsorption on the surface of the catalyst and whether this process leads to ozone decomposition followed by the formation of surface-bound or free radicals (Nawrocki and Kasprzyk-Hordern 2010) and with absorption of light ability. In fact, the presence of UVA with these catalysts photogenerates electrons that can be trapped by ozone reducing to ozonide radical which, in acidic conditions, can be adjuvant on hydroxyl radical production (Mehrjouei et al. 2015; Gomes et al. 2017d, 2018b).

### Kinetic studies

Kinetic studies for applied AOPs were performed for the degradation of each paraben in admixture assuming pseudo-first-order kinetics. The pseudo-first-order kinetic model fitted well with the experimental results with coefficients of determination (*R*^2^) ranging from 0.790 to 0.998. Pseudo-first-order rate constants (*k*′) of the processes are collected in Tables 2, 3 and 4.

**Table 2:** Pseudo-first-order rate constants for paraben mixture phototransformation during O_3_, H_2_O_2_/UVC, H_2_O_2_/O_3_, UVA/O_3_ and H_2_O_2_/O_3_/UVA processes

Process	Paraben	*k*′ (1/min)
O_3_ (8 mg O_3_/L)	MP	0.01737
	EP	0.02295
	PP	0.0245
	BuP	0.01699
	BeP	0.04192
H_2_O_2_/UVC	MP	0.62102
	EP	0.75672
	PP	0.65232
	BuP	0.56989
	BeP	0.88343
H_2_O_2_/O_3_ (70 mg H_2_O_2_/L, 8 mg O_3_/L)	MP	0.02847
	EP	0.02483
	PP	0.02701
	BuP	0.03038
	BeP	0.03128
UVA/O_3_ (8 mg O_3_/L)	MP	0.03111
	EP	0.02938
	PP	0.03151
	BuP	0.02776
	BeP	0.03264
H_2_O_2_/O_3_/UVA (13.5 mg H_2_O_2_/L)	MP	0.036
	EP	0.0363
	PP	0.04206
	BuP	0.03402
	BeP	0.03585

**Table 3:** Pseudo-first-order rate constants for paraben mixture phototransformation during the photocatalytic oxidation under UVA and natural sunlight irradiation

Process	Paraben	Type of catalyst	*k*′ (1/min)
UVA/Cat	MP	Pd-TiO_2_	0.00275
		Pt-TiO_2_	0.00135
		Ag-TiO_2_	0.00347
		Au-TiO_2_	0.00047
		TiO_2_	0.00011
	EP	Pd-TiO_2_	0.00295
		Pt-TiO_2_	0.00148
		Ag-TiO_2_	0.0033
		Au-TiO_2_	0.00053
		TiO_2_	0.000229
	PP	Pd-TiO_2_	0.00336
		Pt-TiO_2_	0.00167
		Ag-TiO_2_	0.00353
		Au-TiO_2_	0.00057
		TiO_2_	0.000226
	BuP	Pd-TiO_2_	0.00377
		Pt-TiO_2_	0.0015
		Ag-TiO_2_	0.00371
		Au-TiO_2_	0.00077
		TiO_2_	0.000813
	BeP	Pd-TiO_2_	0.00751
		Pt-TiO_2_	0.00345
		Ag-TiO_2_	0.00414
		Au-TiO_2_	0.00071
		TiO_2_	0.000544
Sun/Cat	MP	Pd-TiO_2_	0.0404
		Pt-TiO_2_	0.01955
		Ag-TiO_2_	0.00699
		Au-TiO_2_	0.01149
		TiO_2_	0.00158
	EP	Pd-TiO_2_	0.03975
		Pt-TiO_2_	0.01736
		Ag-TiO_2_	0.00731
		Au-TiO_2_	0.01155
		TiO_2_	0.00149
	PP	Pd-TiO_2_	0.03653
		Pt-TiO_2_	0.01692
		Ag-TiO_2_	0.00715
		Au-TiO_2_	0.01132
		TiO_2_	0.0014
	BuP	Pd-TiO_2_	0.03452
		Pt-TiO_2_	0.01658
		Ag-TiO_2_	0.00702
		Au-TiO_2_	0.01113
		TiO_2_	0.00132
	BeP	Pd-TiO_2_	0.03241
		Pt-TiO_2_	0.01597
		Ag-TiO_2_	0.00685
		Au-TiO_2_	0.01079
		TiO_2_	0.00119

**Table 4:** Pseudo-first-order rate constants for paraben mixture phototransformation during the photocatalytic oxidation under UVA and natural sunlight irradiation

Process	Paraben	Type of catalyst	*k*′ (1/min)
O_3_/Cat	MP	Pd-TiO_2_	0.03345
		Pt-TiO_2_	0.03507
		Ag-TiO_2_	0.0204
		Au-TiO_2_	0.01832
		TiO_2_	0.02372
	EP	Pd-TiO_2_	0.03071
		Pt-TiO_2_	0.03395
		Ag-TiO_2_	0.0187
		Au-TiO_2_	0.01641
		TiO_2_	0.02136
	PP	Pd-TiO_2_	0.03267
		Pt-TiO_2_	0.03409
		Ag-TiO_2_	0.02043
		Au-TiO_2_	0.01801
		TiO_2_	0.02342
	BuP	Pd-TiO_2_	0.02383
		Pt-TiO_2_	0.02019
		Ag-TiO_2_	0.01782
		Au-TiO_2_	0.01811
		TiO_2_	0.02343
	BeP	Pd-TiO_2_	0.05988
		Pt-TiO_2_	0.03812
		Ag-TiO_2_	0.03639
		Au-TiO_2_	0.03453
		TiO_2_	0.04889
UVA/Cat/O_3_	MP	Pd-TiO_2_	0.04346
		Pt-TiO_2_	0.03698
		Ag-TiO_2_	0.03925
		Au-TiO_2_	0.02933
		TiO_2_	0.03886
	EP	Pd-TiO_2_	0.04258
		Pt-TiO_2_	0.03443
		Ag-TiO_2_	0.03946
		Au-TiO_2_	0.02716
		TiO_2_	0.03816
	PP	Pd-TiO_2_	0.04542
		Pt-TiO_2_	0.03725
		Ag-TiO_2_	0.04022
		Au-TiO_2_	0.02918
		TiO_2_	0.03506
	BuP	Pd-TiO_2_	0.03978
		Pt-TiO_2_	0.03912
		Ag-TiO_2_	0.03673
		Au-TiO_2_	0.02427
		TiO_2_	0.02993
	BeP	Pd-TiO_2_	0.05755
		Pt-TiO_2_	0.03777
		Ag-TiO_2_	0.05482
		Au-TiO_2_	0.03312
		TiO_2_	0.04214

Firstly, the ozone and hydrogen peroxide process will be discussed (Table 2). The calculated values of pseudo-first-order rate constants for the H_2_O_2_/UVC process for all parabens are 1 order of magnitude higher than those for other processes. It must be mentioned that for this process, the complete removal of each paraben was achieved after just 30 min., while for the H_2_O_2_/O_3_/UVA process, 90 min was required. The rest of the processes (O_3_, H_2_O_2_/UVC, H_2_O_2_/O_3_, UVA/O_3_) needed 2 h or 3 h to remove the paraben concentration at the satisfactory levels. Despite maintaining the same order of magnitude of *k*′, the slight increase in the following order (average values between brackets): O_3_ (0.02475 ± 0.0102 1/min), H_2_O_2_/O_3_ (0.02839 ± 0.0026 L/min), UVA/O_3_ (0.03048 ± 0.0019 L/min) and H_2_O_2_/O_3_/UVA (0.03685 ± 0.0031 L/min) can be observed. As can be concluded from the *k*′ values, the synergetic effect can be seen when the H_2_O_2_/O_3_/UVA process was used, but considering COD removal (Fig. 2), the benefits are not predominant in comparison to the H_2_O_2_/O_3_ process.

When the photocatalytic oxidation process is considered (Table 3), it can be seen that the calculated values of pseudo-first-order rate constants for the processes under sunlight irradiation are 1 order of magnitude higher than those for the process under UVA conditions. The exceptions are TiO_2_-Ag and TiO_2_-Au. For TiO_2_-Ag, the *k*′ is, on average, twice as high using the natural sun in comparison with UVA (0.00706 ± 0.00017 L/min and 0.00363 ± 0.00032 L/min (average values), respectively), while for TiO_2_-Au, the *k*′ determined for photocatalytic oxidation under sunlight condition is 2 orders of magnitude higher (0.01126 ± 0.0003 L/min) than that in UVA condition (0.00061 ± 0.00016 L/min). However, the highest *k*′ values were obtained for Pd-TiO_2_ (0.00407 ± 0.00196 L/min and 0.03672 ± 0.0034 L/min (average values), respectively), UVA/Cat and Sun/Cat processes.

However, when the catalytic ozonation is compared with photocatalytic ozonation, the pseudo-first-order kinetic constants are similar with a slight advantage in favour of the UVA/Cat/O_3_. But when COD removal is taken into account, the implementation of UVA is not reasonable.

In case of all processes, the pseudo-first-order kinetic constants for methylparaben were the lowest while the highest values were obtained for benzylparaben degradation.

### Proposed mechanism

It is known that generally AOPs enable a high degree of degradation of hazardous aqueous contaminants in a short time. Its wide application is connected with •OH generation in near ambient condition (temperature and pressure). The •OH is one of the most powerful oxidants and the most commonly used in water treatment processes, due to its non-selectivity (Glaze et al. 1987). As presented above, many processes are qualified as AOP. The well known are processes involving combinations of O_3_, H_2_O_2_, UV, TiO_2_ and Fenton reagent. The mechanism of those processes is widely investigated. In the H_2_O_2_/UVC system, the cleavage of H_2_O_2_ with UVC light produces a quantum yield of two ^•^OH radicals per unit of radiation absorbed (Eq. (1)). However, the *ε*_254_ value of H_2_O_2_ is very low (19.6 M^−1^/cm) (Glaze et al. 1987), which determines its very high concentration to produce sufficient ^•^OH. Moreover, as can be seen in Fig. 1b, H_2_O_2_ is able to absorb photons below 300 nm, which excludes the UVA lamps.

1 $$ {\mathrm{H}}_2{\mathrm{O}}_2+ hv\leftrightarrow {2}^{\bullet}\mathrm{OH} $$

The Fenton process overcomes these drawbacks because the oxidation processes utilise the activation of H_2_O_2_ by iron salts which allows the generation of ^•^OH (Eq. (2)). Moreover, Fe^3+^ can react with H_2_O_2_ and hydroperoxyl radical (so-called ‘Fenton-like reaction’), which leads to Fe^2+^ regeneration (Eq. (3))

2 $$ {\mathrm{Fe}}^{2+}+{\mathrm{H}}_2{\mathrm{O}}_2\to {\mathrm{H}\mathrm{O}}^{-}+{}^{\bullet}\mathrm{O}\mathrm{H}+{\mathrm{Fe}}^{3+} $$

3 $$ {\mathrm{Fe}}^{3+}+{\mathrm{H}}_2{\mathrm{O}}_2\to {{\mathrm{H}\mathrm{O}}_2}^{\bullet }+{\mathrm{H}}^{+}+{\mathrm{Fe}}^{2+} $$

**Fig. 5:**
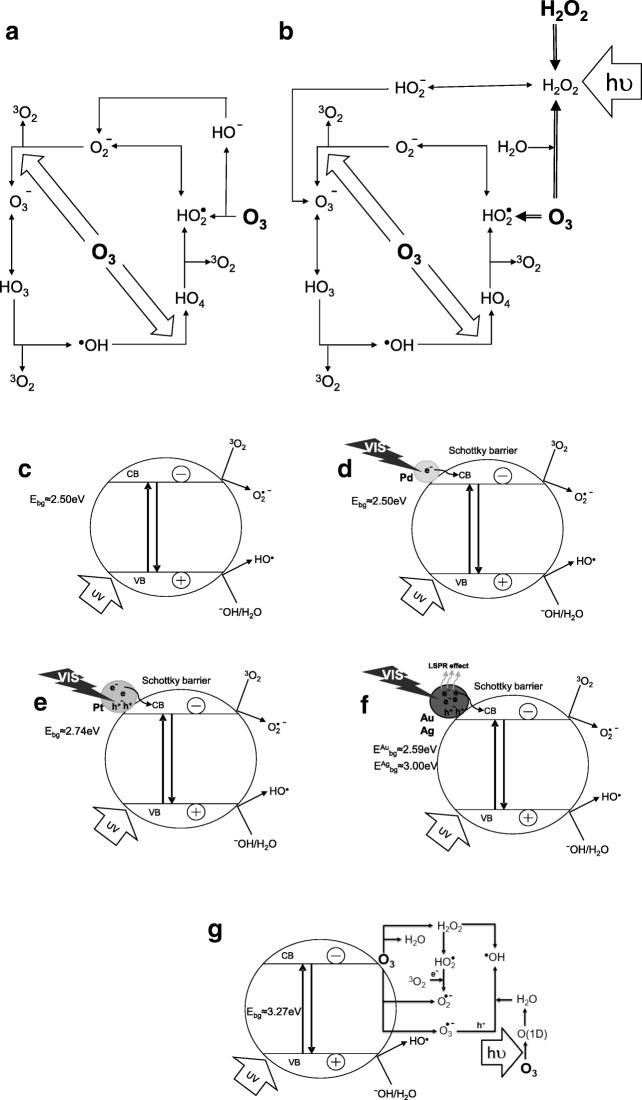
Schematic illustration of the proposed reaction mechanism for ozone-based processes (**a**, **b**), photocatalytic oxidation for TiO_2_ (**c**) and noble metals (**d**–**f**) as well as photocatalytic ozonation (**g**) [based on Glaze 1986, Glaze et al. 1987, Bumajdad and Madkour 2014, Zielińska-Jurek 2014 and Xiao et al. 2015]

The reaction based on O_3_ is more complicated. Firstly, the mechanism of its action is strongly dependent on pH (as discussed above): at high pH values, ozone acts not just by direct reaction but also by formation and reaction of ^•^OH. The decomposition of ozone in a cyclic chain process can be indicated by hydroxide ion (Fig. 5a), H_2_O_2_ supplementation to the system or photolysis of ozone (Fig. 5b). Moreover, despite the two pathways, both the formations of hydrogen peroxide can occur, which can initiate chain decomposition of ozone, resulting in ^•^OH formation (Glaze 1986). Therefore, even reactions that are performed below or near neutral pH may have a component of radical character, because peroxide is often a by-product of the ozonolysis (Glaze 1986). Moreover, the stoichiometric yield of ^•^OH is greater from the photolysis of H_2_O_2_ (only 0.5 mol H_2_O_2_ is consumed for 1 mol of ^•^OH production) in comparison to the photolysis of ozone (1.5 mol O_3_ is consumed for 1 mol of •OH production (and at the same time, 0.5 mol H_2_O_2_ is formed in situ)) at the same UVC fluence dose (Glaze et al. 1987). When O_3_/H_2_O_2_ is considered, only 1 mol O_3_ and 0.5 mol H_2_O_2_ are required to form 1 mol ^•^OH (Glaze et al. 1987). However, taking into account the absorption coefficients, the higher amount of ^•^OH is formed during O_3_ photolysis (2 radicals per incident photon) than H_2_O_2_ photolysis (0.09 radicals per incident photon). Therefore, O_3_/H_2_O_2_ and O_3_/H_2_O_2_/UV have higher effectivity and are more cost-effective (Lucas et al. 2010).

It is known that in the TiO_2_ photocatalytic process, the photocatalytic activity is exhibited by photogenerated charge carriers which is a consequence of the TiO_2_ activation by radiation with energy higher than its band gap energy. Positive holes (*h*^+^) are responsible for oxidising organic compounds due to reactions with a water molecule or OH ions and ^•^OH radicals (Gmurek et al. 2017), while negative electrons (*e*^−^) for reducing molecular oxygen to superoxide radical anions (Fig. 5c). Moreover, the generation of H_2_O_2_ from the electroreduction of oxygen occurred that may also lead to the production of •OH radicals (Salvador 2007; Xiao et al. 2015), while modification of TiO_2_ by a noble metal changed the mechanism of electron transfer. The photons are absorbed by semiconductor (TiO_2_) and/or also by metal nanoparticles through their LSPR excitation (mostly in case of Ag and Au) (Bumajdad and Madkour 2014; Zielińska-Jurek 2014; Wysocka et al. 2018); see Fig. 5d–f. The enhancement in photoactivity is attributed to Schottky barriers under UV range radiation and to LSPR that induces a collective coherent oscillation of the conducting band electrons of the metal nanoparticles. However, it should be mentioned that under UV irradiation (where energy is much higher than the surface plasmon resonance (SPR), the improvement in photoactivity is not related to SPR (Bumajdad and Madkour 2014). However, under visible range, the enhancement is strongly related with LSPR and Schottky barrier (Wysocka et al. 2018; Borowska et al. 2019). Moreover, the electron transfer through the noble metal/TiO_2_ interface under a wide range (UVA and visible) occurred (Hu and Bürgi 2012). The differences between ozone-based or hydrogen peroxide–based AOP and photocatalysis with semiconductors modified by noble metals indicate that photodegradation can be attributed not only to ^•^OH radicals but also to other forms of reactive oxygen species (ROS) ${\mathrm{O}}_2^{\bullet \kern0.5em -}$, ^1^O_2_ and H_2_O_2_ or *e*_cb_^−^ and *h*^+^ (Wysocka et al. 2018).

The addition of ozone significantly enhances the mineralisation prior to heterogeneous photocatalysis alone or even in case of catalysis. This synergetic effect is due to ready hydroxylation of the semiconductor catalyst in aqueous solution (Xiao et al. 2015). Heterogeneous photocatalytic ozonation is a very complicated system that involves chemical, catalytic and photocatalytic reactions. The improvement of the mineralisation during simultaneously occurring photocatalysis and ozonation synergistically intensified the formation of free radicals (Fig. 5g).

Based on the experiments of scavengers, the main degradation pathways via ^•^OH were proved for H_2_O_2_/Fe^2+^, H_2_O_2_/UVC, O_3_/H_2_O_2_, O_3_/UVA, O_3_/H_2_O_2_/UVA, UVA/Cat, O_3_/Cat and O_3_/UVA/Cat. It is known that oxidation of aqueous water contaminants can be proceeded via direct oxidation (the reaction of free holes/electrons with adsorbed contaminates) or indirect oxidation (through oxidation by ROS). However, in photocatalytic processes in which the hydroxyl radicals were confirmed as the main oxidants, the other ROS present in the system (such as ozone, ozonide and superoxide radicals) have a lower effect on paraben degradation and by-product formation and further decay. As a main transformation by-product, *p*-hydroxybenzoic acid, 2,4-dihydroxybenzoic acid and 3,4-dihydroxybenzoic acid as well as monohydroxylated parabens were identified and confirmed the degradation pathway during AOP treatment accoutred predominantly via hydroxylation (Fig. S6).

### Mineralisation, biodegradability and toxicity assessment

**Fig. 6:**
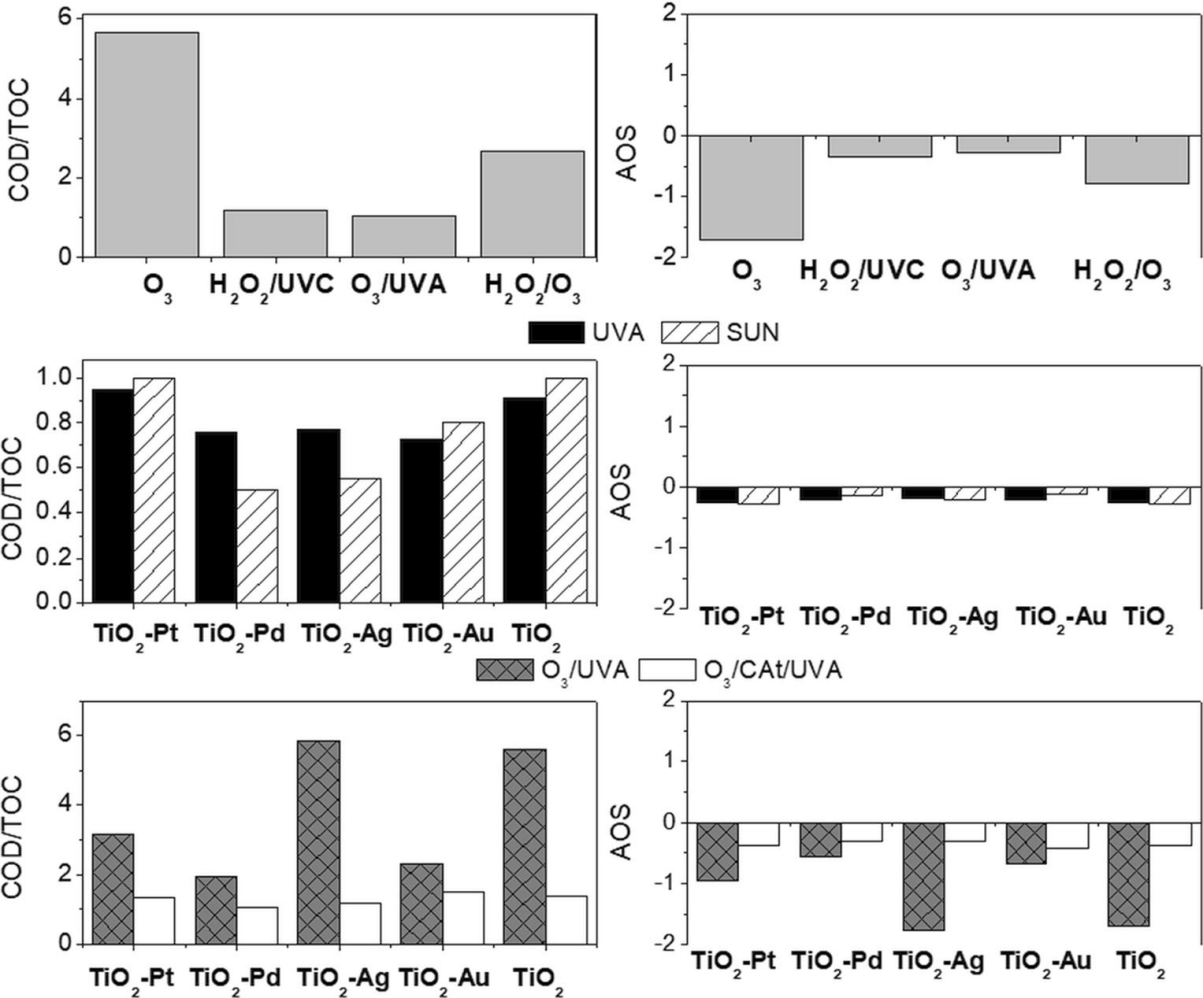
Biodegradability assessment of COD/TOC and AOS *B* values obtained for the decomposition of paraben mixture after several AOPs

For the tested AOPs, the degree of mineralisation was assessed. As discussed above, the mineralisation was expressed by COD. However, for a deeper analysis, total organic carbon (TOC) removal (after 2 h) will be discussed as well (Table S3). Between 80% and 90% COD reduction and 50% TOC removal were observed after 60 min of treatment. As discussed above, the highest COD removal was obtained when H_2_O_2_/UVC was used, which also corresponded with the highest TOC removal. Very good COD reduction was observed after O_3_/H_2_O_2_ (70%), while TOC removal was equal to 26%. For all catalytic processes, the highest TOC reduction was achieved when TiO_2_-Pd was used: UVA/Cat (25%), Sun/Cat (34%), O_3_/Cat (18%) and UVA/O_3_/Cat (38%). In case of UVA/Cat, Sun/Cat and O_3_/Cat, there were no significant differences between the other photocatalysts. The application of UVA/O_3_/Cat process caused the highest COD as well as TOC removal (Table S3). The TOC and COD analysis showed that the total mineralisation was not achieved in any of the processes. It can be suspected that organic transformation by-products are still present in solution after treatments. Therefore, it is necessary to investigate the biodegradability as well as the toxicity of the solution after the treatment. The biodegradability study was based on the investigation of changes in the degree of oxidation, which is an indicator of the oxidation degree of complex solutions, providing indirect information on their probability of biodegradation. The COD/TOC values and average oxidation state (AOS) were used for biodegradability evaluation (Al Momani et al. 2004). The results are shown in Fig. 6.

The initial solution containing a mixture of parabens was characterised by COD/TOC factor equal to 2.91. It should be mentioned that lower COD/TOC ratios imply a higher degree of oxidation while higher ratios mean lower oxidation. As can be seen in Fig. 6, the much higher value of that parameter was obtained after single ozonation, which confirmed that a lot of complexed transformation by-products were present after this treatment. In case of catalytic processes, it seems that when just photocatalytic oxidation or photocatalytic ozonation occurred, the complexity of transformation by-products decreased, despite high differences between COD and TOC removal, while when the catalytic ozonation was proceeded, the COD/TOC is higher than that for initial solution. Moreover, for TiO_2_ and TiO_2_-Ag, the values of that parameter were similar to single ozonation.

It is known that AOS is a valuable parameter that can be used as a general oxidation measure of complex mixtures containing various oxidation products. Theoretically, AOS can take values between + 4 for CO_2_ (the most oxidised state of C) and − 4 for CH_4_ (the most reduced state of C). For example, the AOS for benzene is − 1, that for formaldehyde and acetic acids is 0 while that for formic acid and oxalic acid is + 2 and + 3, respectively (Al Momani et al. 2004). As can be seen in Fig. 6, almost all of the tested AOPs resulted in significant increases in the AOS values (initial AOS = − 2.27). The AOS values after H_2_O_2_/UV, O_3_/UVA, photocatalytic oxidation (both UVA and Sun) and photocatalytic ozonation confirmed that more oxidised by-products are obtained. However, it is still not as simple as carboxylic acids. The lowest oxidation degree was achieved in the case of single ozonation or catalytic oxidation when TiO_2_ and TiO_2_-Ag were used, confirming results from COD/TOC parameter.

As can be seen in Table 5 and as discussed above, almost all processes led to complete degradation of paraben concentration after 120 min. Moreover, when some processes have been applied, the removal of each paraben was achieved much faster. Only during photocatalytic oxidation the degradation after 3 h was incredibly low, and the decontamination has been influenced by the photocatalysts. For all effluents (containing paraben mixture) before treatment, the initial COD value as well as *Aliivibrio fischeri* luminescence inhibition (LI) after 15 min of exposure were 102 ± 7.5 mg O_2_/L and 95.57 ± 0.04%, respectively.

**Table 5:** Maximum paraben concentration, COD removal, *A. fischeri* luminescence inhibition (15 min of exposure (LI^15 min^)), *C. fluminea* mortality and *L. sativum* germination index (GI) after 120 min of treatment

Process	Max. *C*^parabens^	COD (%)	LI^15 min^	Mortality	GI (%)
H_2_O_2_/Fe^2+^*	100%	40	Nd^#^	37%	
H_2_O_2_/UVC*	100%	41	Nd^#^		
UVA/TiO_2_^¥^	12%^BuP^	10	> 80%	ab/p	(43 ± 0)
UVA/TiO_2_-Pt^¥^	42%^BeP^	18	> 80%	55%	(57 ± 23)
UVA/TiO_2_-Pd^¥^	73%^BeP^	19	> 80%	29%	(70 ± 15)
UVA/TiO_2_-Ag^¥^	50%^All^	17	> 80%	21%	(61 ± 9)
UVA/TiO_2_-Au^¥^	16%^BuP^	8	> 80%	ab/p	(44 ± 5)
O_3_/H_2_O_2_	100%	70	57.1 ± 2.8	29%	
O_3_/UVA	100%	27	59.9 ± 1.8	0%	(93 ± 11)
O_3_/TiO_2_	100%	28	26.5 ± 0.5	21%	(71 ± 10)
O_3_/TiO_2_-Pt	100%	35	32.9 ± 1.0	0%	(86 ± 4)
O_3_/TiO_2_-Pd	100%	35	33.7 ± 1.1	15%	(90 ± 3)
O_3_/TiO_2_-Ag	100%	35	35.2 ± 3.9	0%	(80 ± 1)
O_3_/TiO_2_-Au	100%	28	31.4 ± 1.4	0%	(71 ± 5)
O_3_/UVA/TiO_2_	100%	38	36.3 ± 0.0	0%	(90 ± 0)
O_3_/UVA/TiO_2_-Pt	100%	49	61.4 ± 1.2	0%	(107 ± 11)
O_3_/UVA/TiO_2_-Pd	100%	41	44.2 ± 1.4	0%	(108 ± 7)
O_3_/UVA/TiO_2_-Ag	100%	43	43.4 ± 1.4	0%	(112 ± 1)
O_3_/UVA/TiO_2_-Au	100%	38	55.4 ± 1.0	0%	(107 ± 5)

For photocatalytic oxidation, the maximum removal is indicated (the paraben considered is given as superscript)

*ab/p* abnormal behaviour and/or paralysis of *C. fluminea*, *Nd* not determined

*After 60 min of treatment

^#^The toxicity after the treatment time was too low to inhibit luminescence

^¥^After 180 min of treatment

The best results of COD removal have been obtained when H_2_O_2_/UVC and H_2_O_2_/Fe^2+^ were applied, in spite of the lowest time of treatment (1 h). Contrarily, the worst COD abatement was obtained by photocatalytic oxidation (UVA) when the treatment time was the longest one (3 h). It should be noticed that during this process, no more than 50% removal of each paraben concentration was achieved, while during the other processes, the degradation of parabens was obtained before 120 min.

The initial paraben mixture was highly toxic to *A. fischeri*. During the reaction, the toxicity has been reduced. According to the literature, the sample can be considered non-toxic when LI is below 30% (Miralles-Cuevas et al. 2017). Taking this value into account, for the Fenton process and H_2_O_2_/UVC, non-toxic effluents have been achieved. Similar effect has been obtained for O_3_/H_2_O_2_ and O_3_/UVA (irrespective to COD removal). When the processes with photocatalysts were considered, large differences have been noticed. It was found that the photocatalytic oxidation gave the worst results (LI^15 min^ > 80%). According to the literature when LI is higher than 80%, the bioassay test is not sensitive to changes, which indicates still very high toxicity towards *A. fischeri* (Miralles-Cuevas et al. 2017). Catalytic ozonation gives much better toxicity results than photocatalytic ozonation, despite lower COD removal. This can be related with the by-product production. In fact, Gomes et al. (2019) verified that the presence of hydroxyl radicals for the same paraben mixture in a general way shows more toxicity than the single ozone due to the generation of different by-products.

In the case of Asian clams, for the same conditions, for the initial mixture of parabens, 100% mortality was achieved after 72 h of contact. It has to be mentioned that *C. fluminea* is intolerant at the concentration above 7.5 mg/L of each, and the LC_50_ of the paraben mixture solution is equal to 56% (Gomes et al. 2017a). Therefore, for all treatment processes (photolysis, UVA/TiO_2_ and UVA/TiO_2_-Au) where the degradation was not efficient enough to cause higher concentration, abnormal foot extension and/or paralysis was observed. However, for TiO_2_-Pt, TiO_2_-Ag and TiO_2_-Pd, the reduction of paraben toxicity towards *C. fluminea* after the treatment was observed. While after all catalytic ozonation experiments (except for TiO_2_-Pd- and Pd-TiO_2_), no mortality was observed. For the samples obtained after photolytic ozonation and photocatalytic ozonation with different photocatalysts, no mortality was verified for all concentrations tested after 72 h of exposure. The solution after the Fenton process exhibits higher mortality than O_3_/H_2_O_2_ and even after single ozonation (29%).

The high toxic impact of paraben mixture (GI = (42 ± 11)%) towards *Lepidium sativum* was investigated. According to Trautmann and Krasny (1997), this germination index proves strong or severe inhibition, while according to Wang (1992), the inhibition of the root growth of *L. sativum* can be classified as very strong or strong. Almost all treatments increased the phytotoxicity. The photocatalytic oxidation (UVA/TiO_2_, UVA/TiO_2_-Au, UVA/TiO_2_-Pt) slightly reduced toxicity; however, the inhibition is still strong. After single ozonation (GI = (65 ± 5)%) with UVA/TiO_2_-Pd and UVA/TiO_2_-Ag and catalytic ozonation with TiO_2_, TiO_2_-Au and TiO_2_-Ag (O_3_/TiO_2_ and O_3_/TiO_2_-Ag), just mild inhibition is observed, which means moderate phytotoxicity. While after O_3_/H_2_O_2_ and photocatalytic ozonation with all photocatalysts, no inhibition of plant growth was achieved.

### UV dose and cost-effectiveness

**Fig. 7:**
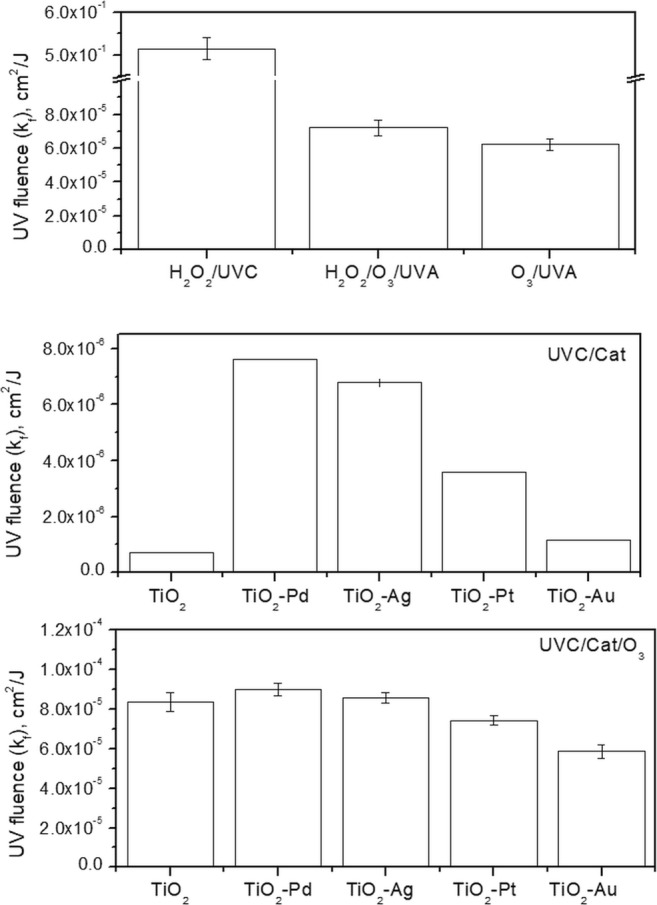
The obtained pseudo-first-order fluence-based rate constants for paraben mixture degradation by various AOPs

Kinetics was also investigated from the UV fluence point of view (Fig. 7). Following these, the pseudo-first-order degradation rate constants for UV processes were also determined for the curves ln(*C*/*C*_0_) (as an average for all paraben degradations) vs. fluence (not shown), revealing linear correlation coefficients of 0.92–0.98.

It can be seen that the H_2_O_2_/UVC process which was the most effective in paraben decomposition is also the least time- and energy-consuming process. The application of UVA (8.9 W/m^2^) instead UVC (31.8 W/m^2^), despite the lower lamp power, decreased dramatically *k*_f_. Even additional oxidant (O_3_) did not cause the comparable degradation values, proving that H_2_O_2_/O_3_/UVA and O_3_/UVA are much more energy-consuming. The highest value was obtained for TiO_2_-Pd [(7.63 ± 3.69) × 10^−6^ cm^2^/J and (8.96 ± 0.34) × 10^−5^ cm^2^/J], for photocatalytic oxidation processes and photocatalytic ozonation, respectively. Also for both processes, TiO_2_-Ag was very effective [(6.80 ± 0.59) × 10^−6^ cm^2^/J for UVA/TiO_2_-Ag and (8.56 ± 0.27) × 10^−5^ cm^2^/J for UVA/TiO_2_-Ag/O_3_]. For the rest of photocatalysts, the behaviour was different for the case of UVA/Cat and UVA/Cat/O_3_. When the photocatalytic oxidation was performed, the *k*_f_ value decreases in the following order: TiO_2_-Pt ((3.58 ± 1.63) × 10^−6^ cm^2^/J) > TiO_2_-Au ((1.14 ± 0.23) × 10^−6^ cm^2^/J) > TiO_2_ ((0.72 ± 0.07) × 10^−6^ cm^2^/J), while for that system where O_3_ was supplemented, the UV fluence–based pseudo-first-order rate constant for TiO_2_ ((8.35 ± 0.49) × 10^−5^ cm^2^/J) was higher than that for TiO_2_-Pt ((7.40 ± 0.23) × 10^−5^ cm^2^/J) > TiO_2_-Au ((5.84 ± 0.34) × 10^−5^ cm^2^/J). These results indicate that UVA/Cat processes are the most energy-consuming process. Moreover, it was shown that the application of UVA not only increases the time of treatment decreasing mineralisation, which leads to higher energy consumption which determines the treatment costs.

If the application of these methods is to be considered in actual wastewater treatment, the energy cost as well as the operational cost must be known. In this regard, specific energy consumption (SEC) which defines the amount of electrical energy consumption (kWh) per unit mass of COD was calculated (Saien et al. 2014; Shen et al. 2017). When the process was based on the photochemical reaction, Eq. (4) was used (Saien et al. 2014), while in O_3_-based processes, SEC was calculated according to Eq. (5) (Shen et al. 2017).

4 $$ \mathrm{SEC}=\frac{P\times t}{V\times \left({\mathrm{COD}}_0-{\mathrm{COD}}_t\right)} $$

5 $$ {\mathrm{SEC}}_{{\mathrm{O}}_3}=\frac{P\times t+r\times \mathrm{TOD}}{V\times \left({\mathrm{COD}}_0-{\mathrm{COD}}_t\right)} $$

where *P* is the nominal electric power (kW) of the photochemical system, *t* is the reaction time (h), *r* is the energy requirement for O_3_ production (15 kW h/kg O_3_) (Katsoyiannis et al. 2011) and *V* is the volume (L) of the solution in the reactor.

The cost of a treatment from the electricity point of view was found by multiplying the cost of electricity with the SEC value. In the total operational costs of a treatment, several factors were included: oxidants costs, operating costs for ozone production (1.97 €/kg O_3_ (Ried et al. 2009) and costs of catalyst (synthesis and the metal modification).

**Fig. 8:**
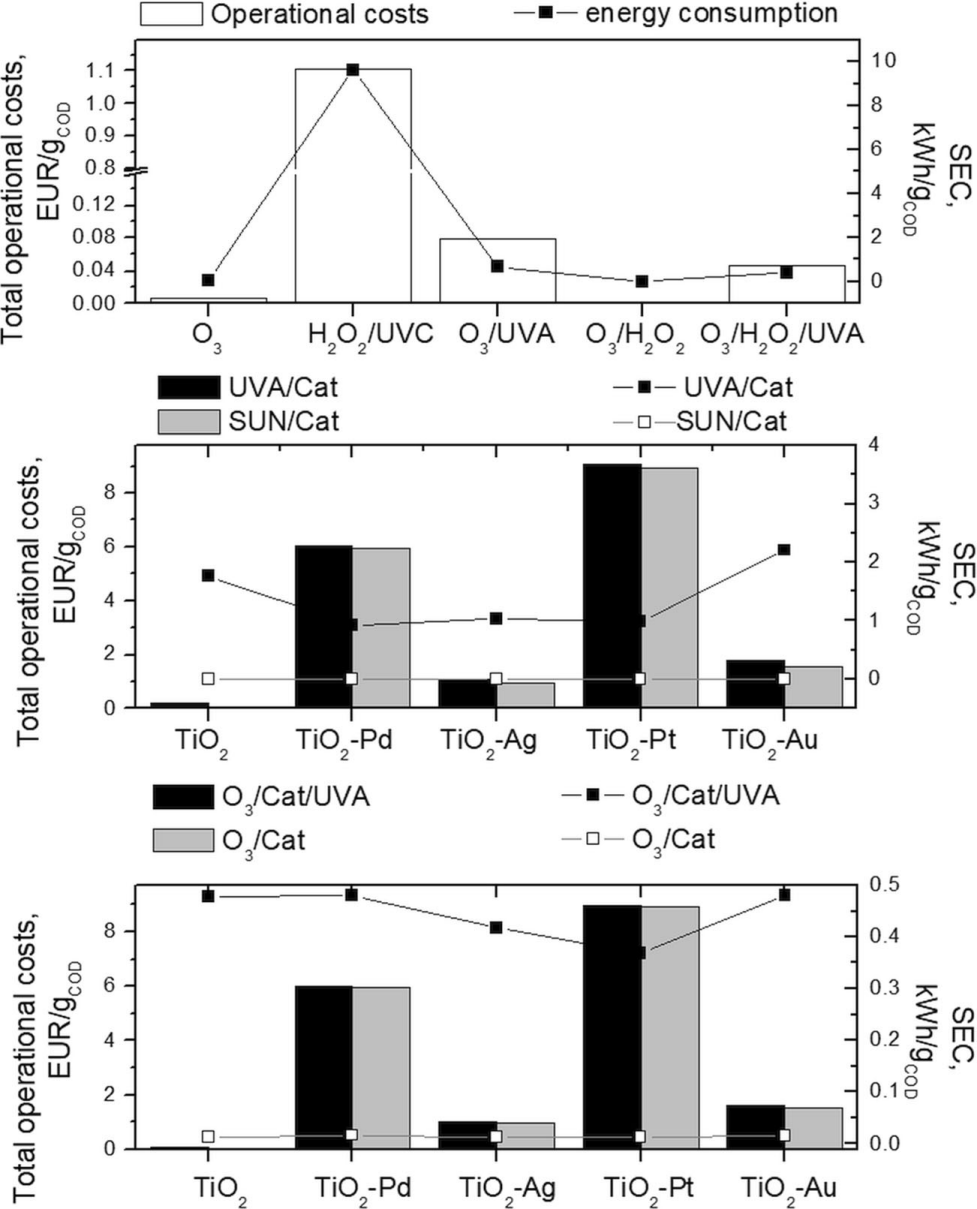
Total operational costs and specific energy consumption for the applied processes (in lab scale)

It can be seen that the lowest SEC was calculated for O_3_, O_3_/H_2_O_2_ and Cat/O_3_ due to the lack of requirement of light source application, while for processes UVA/Cat/O_3_, O_3_/H_2_O_2_ and O_3_/UVA, SEC is 1 order of magnitude lower than that for UVA/Cat and H_2_O_2_/UVC (it is obvious that SEC for Sun/Cat is equal to 0). Despite the UVA energy consumption for the decomposition, the UVC treatment is much higher in energy cost. Besides the cost of electricity, there are also expenses for hydrogen peroxide and ozone production. However, the costs of photocatalyst synthesis (especially the cost of noble metals) are predominant in the catalytic process. Despite the highest efficiency, TiO_2_-Pd is characterised by one of the highest operational costs, due to the Pd price, while for TiO_2_-Ag, the costs are positioned around 197 €/g COD. In case of those catalysts, the energy consumption is not too high if we consider it from the TOC and COD decrease point of view. The results indicate that the use of 0.42 kWh/g COD causes 37% and 43% of TOC and COD decrease, respectively (Fig. 8, Table S3), which provides a significant and attractive advantage.

## Conclusions

The comparison of several radical-driven technologies for mixture degradation of five parabens was presented. Several AOP methods were examined to degrade parabens from aqueous environment as well as to decrease its toxicity. The ozonation in different process parameters was discussed, bearing in mind that combining ozone with H_2_O_2_, UVA as well as noble metal modified TiO_2_. The pseudo-first-order kinetic constants (based on time and on UV fluence) were determined. The biodegradability and toxicity assessment has been presented, considering the toxic effect over several species.

For all applied processes, the same trend was observed. The BeP was the fastest degraded paraben, then followed in the order BuP > PP > EP > MeP. The treatments reduced not only COD but also toxicity towards *V. fischeri*, *C. fluminea* and *L. sativum*, despite the fact that the total COD removal was not achieved. This indicates that the refractory by-products produced are less toxic than parabens.

These results can indicate that the degradation pathway is different, despite the hydroxyl radicals are the main oxidant. The next explanation is that the same TPs formed at the last stage of degradation are in higher concentration. This finding can be with agreement that lower COD removal gives better toxicity results. However, it should be also noticed that the application of pure TiO_2_ leads to lower toxicity and lower COD removal in comparison to photocatalysts modified by noble metals. Furthermore, from all metals used for TiO_2_ modification, platinum seems to be the best. Moreover, it is evident that the application of ozone not only improves detoxification, but also higher mineralisation is observed after O_3_-based treatment.

The results reveal that the treatment efficiency as well as the costs largely depend on the implemented AOP. Nevertheless, the application of hydrogen peroxide, ozone with second oxidant (UVA or H_2_O_2_) and TiO_2_ modified by a noble metal led to much short depletion times when compared with photolysis. Moreover, the synergetic effect not only improved mineralisation and biodegradability but also reduced toxicity and energetic costs.

However, the application of natural sunlight for the photocatalytic oxidation process reduced the cost of lamp equipment as well as electricity, but taking into account the highest mineralisation and biodegradability, the most appropriate cost-effective treatment seems to be photocatalytic ozonation with a UVA lamp. Moreover, detailed characterisation of photocatalysts showed that surface modification of TiO_2_ with noble metals significantly improved the absorption properties of the catalyst resulting in higher COD abatement from aqueous solutions compared to pure TiO_2_. This statement was also supported by the width of the band gap of the photocatalysts. The most promising photocatalysts seem to be TiO_2_ modified by Pd nanoparticles, due to the highest band gap and highest mineralisation. However, when the total operating cost will be taken into account, more reasonable is to use the TiO_2_-Ag that gives satisfactory mineralisation.

## Electronic supplementary materials

ESM 1 (PDF 1077 kb).
