# Supplementary material for: Comparison of radical-driven technologies applied for paraben mixture degradation: mechanism, biodegradability, toxicity and cost assessment
Source: Environ Sci Pollut Res Int. 2019 Nov 20;26(36):37174–92. doi: 10.1007/s11356-019-06703-9 (PMC6937227; doi:10.1007/s11356-019-06703-9)
Supplement: Supplementary file 1 — (PDF 1077 kb). [file 11356_2019_6703_MOESM1_ESM.pdf]

# Supplementary information

## Comparison of radical driven technologies applied for parabens mixture degradation

***Marta Gmurek**[1,2], João F. Gomes[1], Rui C. Martins[1], Rosa M. Quinta-Ferreira[1]*

*(1) CIEPQPF—Chemical Engineering Processes and Forest Products Research Center, Department of Chemical Engineering, Faculty of Sciences and Technology, University of Coimbra, Coimbra, Portugal.*

*(2) Faculty of Process and Environmental Engineering, Department of Bioprocess Engineering, Lodz University of Technology, Lodz, Poland.*
*marta.gmurek@p.lodz.pl*

SI content

Figure:
Fig S1 Schemes of photoreactors (a) UVC experiments (the working volume of each tube 0.01L, external lamps source), (b) UVA experiments as (the working volume 2L, internal lamps source), (c) sunlight experiments (the working volume 0.5L, external light source)
Fig S*2* Removal of each paraben concentration during parabens mixture photolysis under UVC, UVA and natural Sunlight irradiation
Fig S3 COD abatement as a function of TOD for ozone based technologies
Fig. S4. Tauc plot - determination of the direct and indirect bandgaps
Fig.S5 SEM images of photocatalysts (a) $TiO_2$; b) $TiO_2$-Ag; c) $TiO_2$-Pt; d) $TiO_2$-Au; e) $TiO_2$-Pd)
Fig.S6. Possible pathway of the first stage photohydroxylation of parabens during AOPs treatment

Table:
Tab. S1. Process parameters for all studied AOPs
Tab S2. XRD characterization of TiO2-Pd, TiO2-Pt and TiO2-Au photocatalysts
Tab. S3. COD and TOC removal after 2h of treatment

**Figures:**

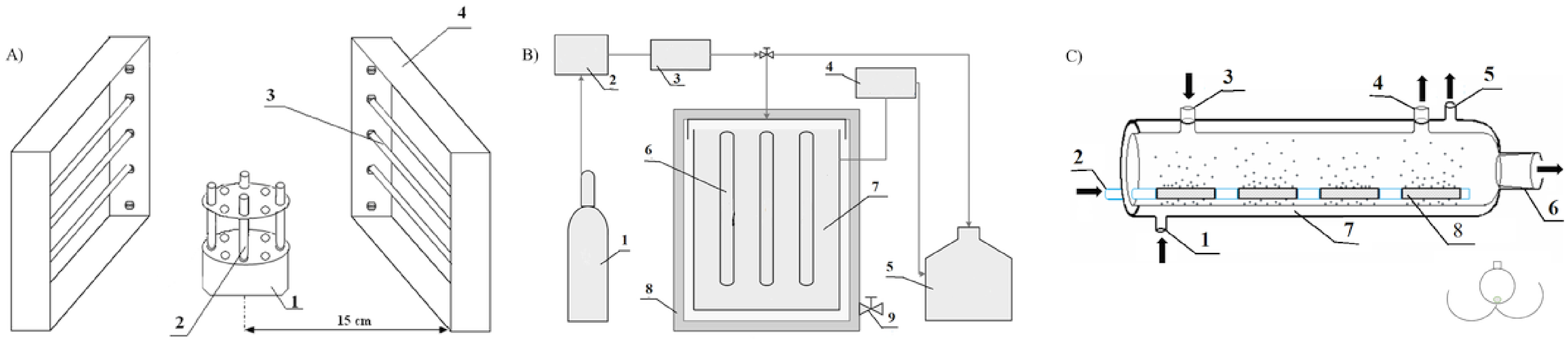


1. merry-go-round device; 2. quartz tube (10mL); 3. UVC lamps; 4. panel with lamps

1. oxygen container; 2. ozone generator; (BMT 802N); 3.,4. ozone analyzers (BMT 963 and 964) ; 5. ozone scavenger; 6. UVA lamps; 7. reactor; 8. cooling jacket; 9. Sampling place

1. inlet of cooling water; 2.air inlet; 3. inlet for reaction mixture, sampling; 4. smapling place and outlet of air; 5. outlet of cooling water; 6. valve for emptying the reactor; 7. cooling jacket; 8. ceramic-glass diffuser

Fig S2 Schemes of photoreactors (a) UVC experiments (the working volume of each tube 0.01L, external lamps source), (b) UVA experiments as (the working volume 2L, internal lamps source), (c) sunlight experiments (the working volume 0.5L, external light source)

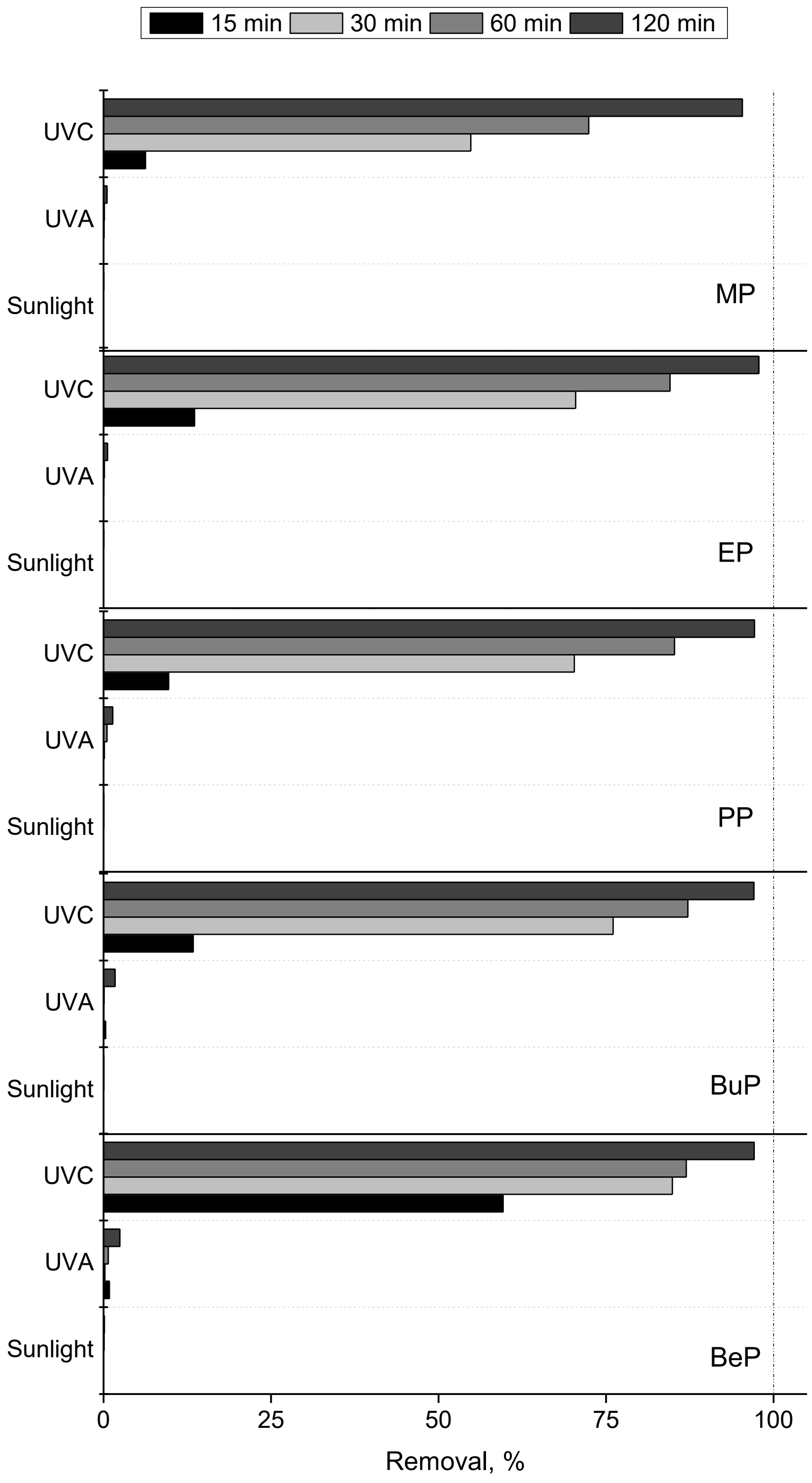


Fig S2 Removal of each paraben concentration during parabens mixture photolysis under UVC, UVA and natural Sunlight irradiation

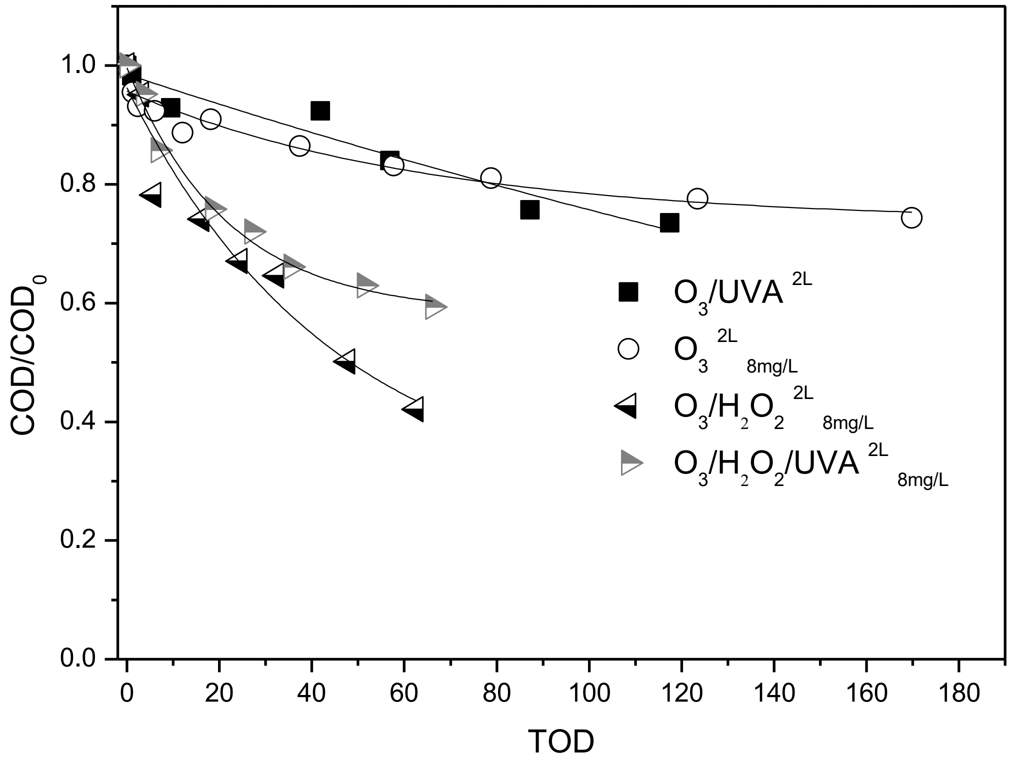


Fig S3 COD abatement as a function of TOD for ozone based technologies

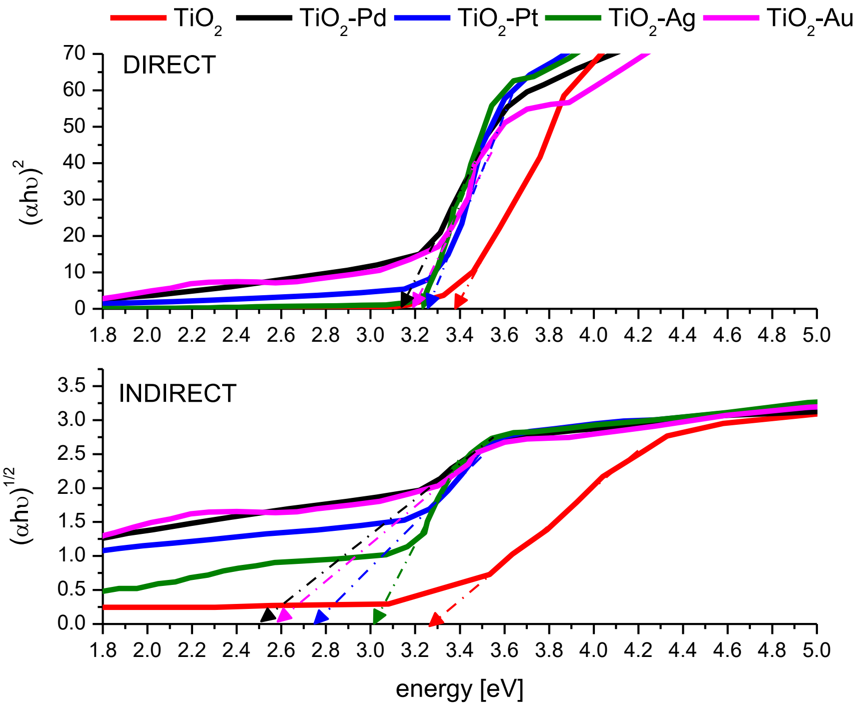


Fig. S4. Tauc plot - determination of the direct and indirect bandgaps

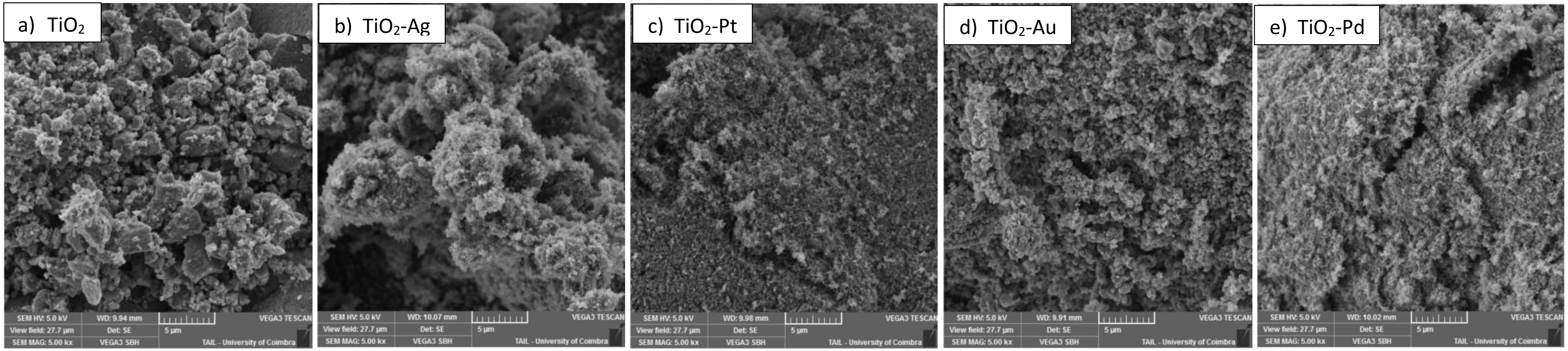


Fig.S5 SEM images of photocatalysts (a) $TiO_2$; b) $TiO_2$-Ag; c) $TiO_2$-Pt; d) $TiO_2$-Au; e) $TiO_2$-Pd)

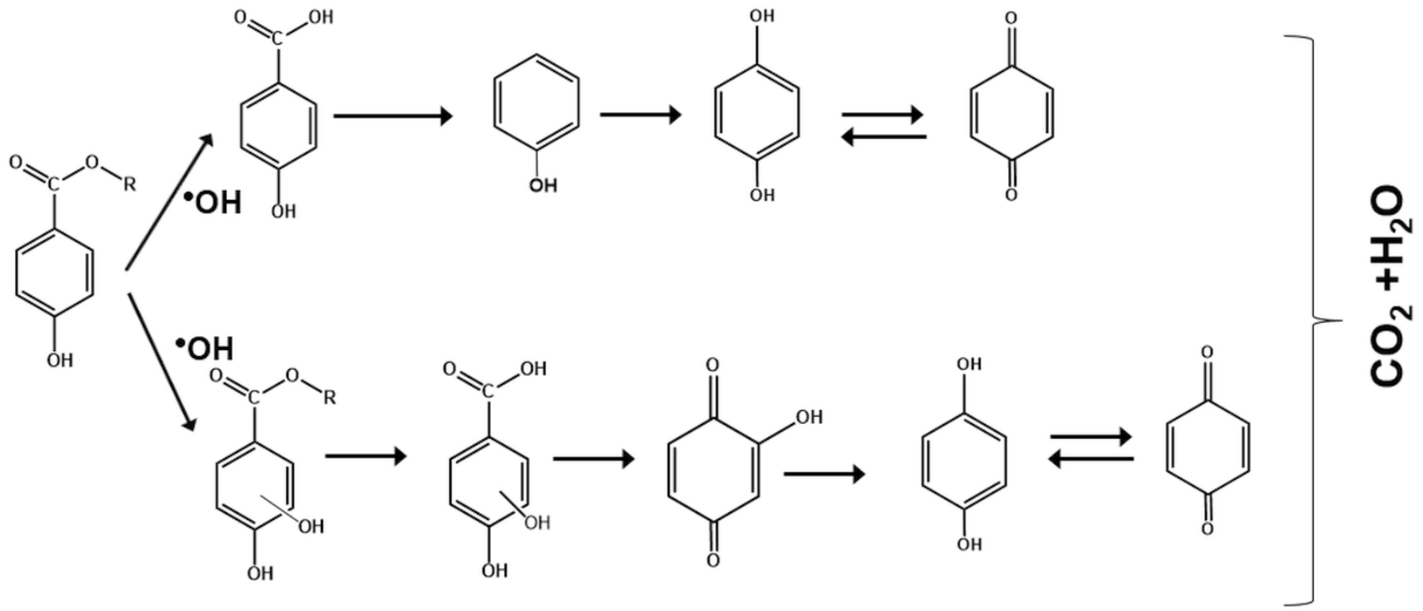


Fig.S6. Possible pathway of the first stage photohydroxylation of parabens during AOPs treatment

## Tables

Tab. S1. Process parameters for all studied AOPs

| Process/symbol | | Condition | | | | | |
|---|---|---|---|---|---|---|---|
| | O3 | H2O2 | Fe2+ | Catalysts | UV photon flux | pH | Reactor volume |
| | mg/L | mg/L | mg/L | mg/L | Einstein/ Ls | | L |
| $O_3$_0.5L | 45 | - | - | - | - | 7[b] | 0.5 |
| | 20 | - | - | - | - | 7 [b] | 0.5 |
| $O_3$_2L | 8 | - | - | - | - | | 2L |
| $O_3/H_2O_2$_0.5L | 45 | 2.5 | - | - | - | w.a.* | 0.5 |
| $H_2O_2$/UVC | - | 1700 | - | - | $1{\times}10^{-5}$ | 7 [b] | 0.01 |
| $H_2O_2/Fe^{2+}$ | - | 300 | 10 | - | - | w.a.[ŧ] | 0.5 |
| $O_3$/ $H_2O_2$_2L | 8 | 70 | - | | | w.a.* | 2 |
| $O_3$/UVA _2L | 8 | - | - | | $5.6{\times}10^{-7}$ | w.a.[$] | 2 |
| $O_3$/UVA /$H_2O_2$ | 8 | 13.5 | - | | $5.6{\times}10^{-7}$ | w.a.* | 2 |
| Cat/UVA | - | - | - | 70 | $5.6{\times}10^{-7}$ | w.a.[$] | 2 |
| Cat/$O_3$ | 8 | - | - | 70 | | w.a.[$] | 2 |
| Cat/$O_3$/UVA | 8 | - | - | 70 | $5.6{\times}10^{-7}$ | w.a.[$] | 2 |

w.a -without adjustment

[b] buffered solution

[ŧ] initial pH= 6±0.7 after addition $H_2O_2$ drop to 3.2

[*] initial pH= 5.7 after addition $H_2O_2$ drop to 3.2

[$] initial pH= 5.7 during the reaction small drop was observed

Tab. S2. Characterization of photocatalysts: XRD analysis, Specific surface area (BET), relative elemental metal concentrations obtained from the XPS analysis

| | Anatase | Rutile | Crystallite size | BET | Ti content | O content | C content | Metal content |
|---|---|---|---|---|---|---|---|---|
| | % | % | nm | $m^2/g$ | at.% | at.% | at.% | at.% |
| $TiO_2$-Pd (0.5% wt.) | 80 | 20 | 203-223 | 53 | 55.88 ± 0.05 | 23.66 ± 0.62 | 20.24 ± 0.66 | 0.23 ± 0.01 |
| $TiO_2$-Pt (0.5% wt.) | 79 | 21 | 198-215 | 53 | 48.70 ± 0.55 | 20.73 ± 0.01 | 30.46 ± 0.54 | 0.13 ± 0.01 |
| $TiO_2$-Au (0.5% wt.) | 99 | 1 | 10-12 | 135 | 14.30 ± 0.07 | 76.20 ± 0.44 | 9.48 ± 0.37 | 0.02 ± 0.00 |
| $TiO_2$-Ag (0.5% wt.) | 80 | 20 | 45-52 | 34 | 58.63 ± 0.66 | 23.84 ± 0.25 | 17.06 ± 0.88 | 0.47 ± 0.04 |

BET for $TiO_2$= 51 $m^2/g$; TIP== 160 $m^2/g$

Tab. S3. COD and TOC removal after 2h of treatment

| | | | | | UVA/Cat | | | | | Sun/Cat | | | | | O3/Cat | | | | | UVA/O3/Cat | | | | |
|---|---|---|---|---|---|---|---|---|---|---|---|---|---|---|---|---|---|---|---|---|---|---|---|---|
| | O3 | H2O2/UVC | O3/UVA | O3/H2O2 | TiO2-Pt | TiO2-Pd | TiO2-Ag | TiO2-Au | TiO2 | TiO2-Pt | TiO2-Pd | TiO2-Ag | TiO2-Au | TiO2 | TiO2-Pt | TiO2-Pd | TiO2-Ag | TiO2-Au | TiO2 | TiO2-Pt | TiO2-Pd | TiO2-Ag | TiO2-Au | TiO2 |
| COD,% | 17 | 80 | 27 | 70 | 18 | 19 | 17 | 8 | 10 | 15 | 17 | 10 | 12 | 10 | 35 | 35 | 35 | 28 | 28 | 49 | 41 | 43 | 38 | 38 |
| TOC, % | 3 | 67 | 26 | 26 | 19 | 25 | 22 | 11 | 11 | 15 | 34 | 18 | 15 | 10 | 11 | 18 | 6 | 12 | 5 | 37 | 38 | 37 | 25 | 28 |
